# Design and validation of nucleosome binding peptides using BindCraft

**DOI:** 10.3389/fbioe.2026.1946220

**Published:** 2026-10-22

**Authors:** Pavel D. Oleinikov, Grigoriy A. Armeev, Alina E. Sidorkina, Elizaveta B. Rykunova, Galina A. Komarova, Nikita A. Motorin, Nadezhda S. Gerasimova, Alexey V. Feofanov, Vasily M. Studitsky, Alexey K. Shaytan

**Affiliations:** 1 Department of Biology, Lomonosov Moscow State University, Moscow, Russia; 2 Department of Physics, Lomonosov Moscow State University, Moscow, Russia; 3 Institute of Gene Biology, Moscow, Russia; 4 Nuclear Dynamics and Cancer Program, Fox Chase Cancer Center, Philadelphia, PA, United States

**Keywords:** AI, chromatin, epigenetics, nucleosome, peptide, protein design

## Abstract

Chromatin is a sophisticated assembly of DNA, RNA and proteins in the nuclei of eukaryotic cells. Protein-protein interactions that govern chromatin function are key regulators of genome activity, making them prime targets for drug discovery. Engineering and modulating these contacts presents a viable pathway for innovative epigenetic therapies. Nucleosomes, the primary structural units of chromatin, feature various interaction interfaces, most notably the acidic patch. This region serves as a critical binding site for numerous chromatin-associated proteins, often via their intrinsically disordered regions. Advances in AI-driven protein design have recently opened new avenues for the rational development of molecules aimed at modulating these chromatin interactions. In this research, we implemented an integrated computational and experimental framework to design and validate peptides targeting the nucleosome acidic patch. We relied on the AI protein design approach implemented in BindCraft to design nucleosome binding peptides. To achieve precise quantitative analysis of these interactions, we refined a donor FRET quenching assay specifically for this application, enabling sensitive and consistent measurement of binding affinities for the designed sequences. We successfully identified peptides with high affinity for the acidic patch on canonical nucleosome core particles. These findings highlight the potential of AI-assisted design for chromatin-targeting agents and establish an effective workflow for their validation, laying the groundwork for synthetic epigenetic modulators and the broader use of protein design in managing 3D genome organization.

## 1 Introduction

Nucleosomes are the fundamental structural units of eukaryotic chromatin, comprising DNA wrapped around a histone octamer. They compact the genome and control access of the epigenetic machinery to DNA, thereby underpinning virtually all nuclear processes. More than three thousand proteins have been associated with chromatin (Lyubitelev et al., 2016; Gribkova et al., 2026), many of which interact with nucleosomes through histone tails or the globular histone core (Horn and Ingen, 2018). Although the overall histone surface is positively charged, it contains a discrete cluster of negatively charged residues, the acidic patch (Barbera et al., 2006) which serves as a key interaction interface for host proteins, pathogen-derived factors (Lagadec et al., 2022), and antibodies (Oleinikov et al., 2023). Well-characterized acidic patch-binding proteins include the N-terminal tail of histone H4, HMGN2 (Teles et al., 2020), CENP-C (Kato et al., 2013), IE1 (Fang et al., 2016), RCC1 (Teles et al., 2020), and LANA (Barbera et al., 2006), several of which show preferential affinity for centromeric nucleosomes. The importance of this region is further highlighted by the recurrent somatic mutations in histone genes mapping to the acidic patch that have been identified in multiple cancer types (Dao and Pham, 2022).

These observations point to nucleosomes not as structural elements, but as regulatory hubs whose interfaces can be targeted for functional intervention. Nucleosome-binding peptides are particularly attractive in the context of serving both as probes for studying protein-nucleosome interactions and as effectors for modulating genome function (Wagner et al., 2020). One compelling example is the synthetic catalyst PEG-LANA-DSSMe, which tethers to the acidic patch and drives regioselective acetylation of histone H2BK120 in living cells, blocking ubiquitination at this site (Fujiwara et al., 2021). In a different application, the CRISPR-Chrom platform fuses Cas9 with chromatin-binding and remodeling peptides to overcome the well-known problem of nucleosome-occluded genomic regions, achieving a three-to six-fold gain in editing efficiency at otherwise inaccessible loci (Ding et al., 2019). Together, these examples illustrate how peptides that engage nucleosomes can act as standalone agents or as functional modules within larger epigenome-editing systems. Designing such peptides from scratch, however, is not straightforward, as the structural repertoire of known peptide-nucleosome interactions remains limited, providing only a small set of templates for a classical rational design. Recent advances in AI-based structure prediction and protein design make it possible to leverage interaction motifs from unrelated protein complexes and engineer nucleosome-binding peptides beyond existing structural examples.

In this study, we used the BindCraft (Pacesa et al., 2025) algorithm which builds on AlphaFold2 (Jumper et al., 2021) to design peptides targeting the H2A–H2B acidic patch of the nucleosome. To test whether the designs actually bind to the nucleosome surface, we developed a dedicated assay for measuring dissociation constants (Kd). Therefore we demonstrate that modern protein design algorithms allow the robust creation of the nucleosome binding peptides even with a relatively small number of experimentally tested candidates (Figure 1). By integrating computational design with experimental validation, this study advances the development of synthetic nucleosome-binding peptides and broadens the possibilities for targeted manipulation of chromatin.

**FIGURE 1:**
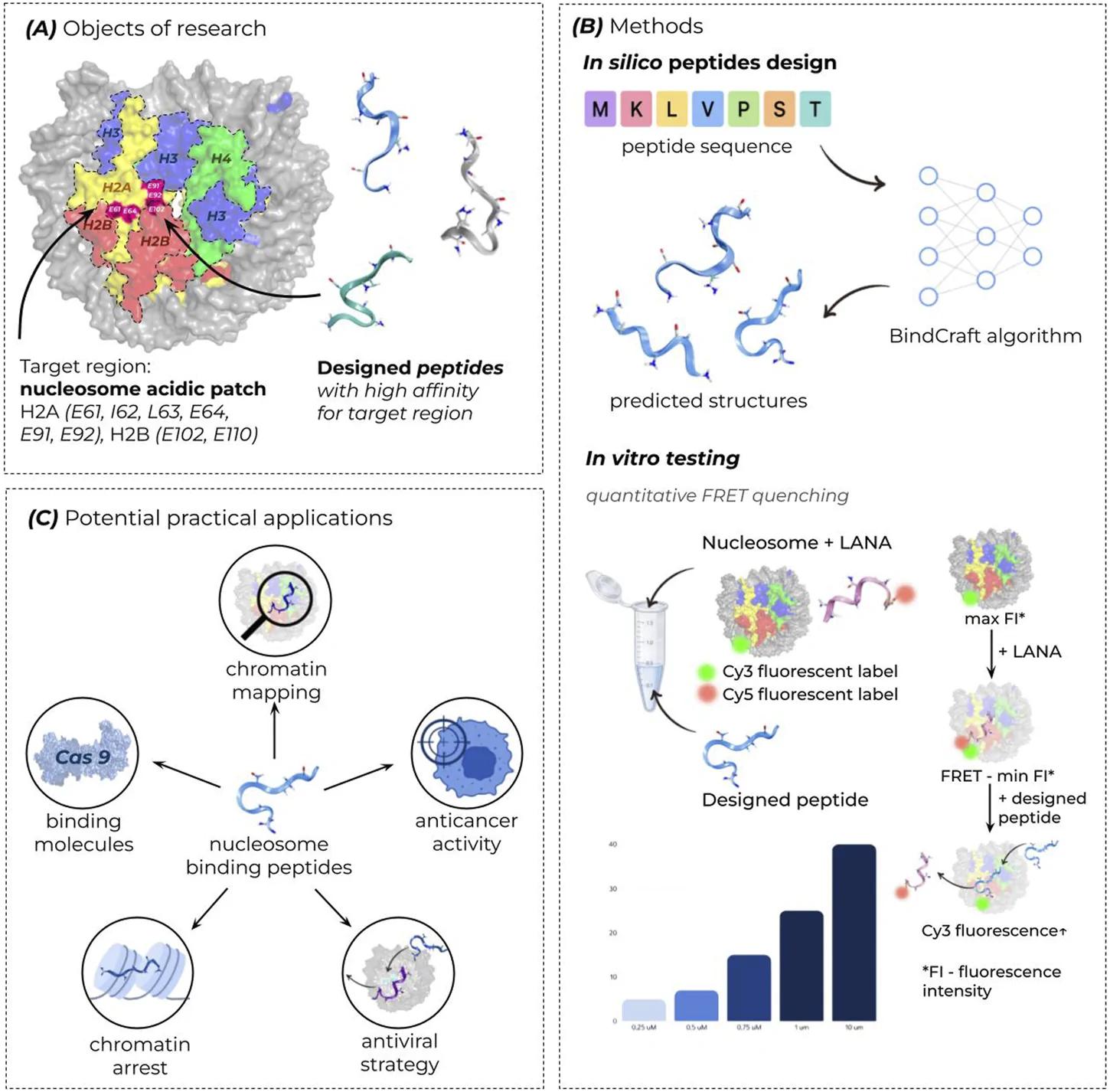
Schematic overview of nucleosome binding peptides research, including **(A)** main objects of study. Left–structure of a whole nucleosome core particle (PDB ID 3LZ0 (Vasudevan et al., 2010)) in the surface representation showing the target binding sites (residues E56, E61, E64, D90, E91, E92 of H2A and E102, E110 of H2B (Kalashnikova et al., 2013)) of designed peptides. The surface is colored according to the conventional histone color scheme: H2A, yellow; H2B, red; H3, blue; and H4, green. Target residues are highlighted in magenta. Right–schematic peptide representations. **(B)** Methodological approaches. Upper panel: *in silico* peptide design using the BindCraft algorithm. Lower panel: *in vitro* validation of the designed peptides using developed quantitative fret quenching assay. **(C)** Potential practical applications of the nucleosome binding peptides.

## 2 Methods

### 2.1 In silico peptide design and candidate selection criteria

Peptide sequences targeting the acidic patch interface of the H2A–H2B dimer (residues E56, E61, E64, D90, E91, E92 of H2A and E102, E110 of H2B (Kalashnikova et al., 2013); PDB: 3LZ0 (Vasudevan et al., 2010)) were generated using the BindCraft algorithm with the peptide_3stage_multimer_flexible protocol (Evans et al., 2021; Li et al., 2023) and sequence length constrained to 15–30 residues. Although there is a Widom 603 DNA sequence containing structure used while experimental validation, for sequences generation we used the Widom 601 DNA sequence containing structure due to its better resolution, the fact that binding site is located on the protein part of the nucleosome core particle and the similarity of NCPs formed on 601 and 603 sequences (Armeev et al., 2025). We used isolated H2A-H2B dimer as DNA structure can not be predicted with AlphaFold2/BindCraft and prediction of histone octamer complexes with peptides was computationally prohibitive. Predictions were performed on the NVIDIA RTX A5000 GPU. 100 sequences were generated; the top 40 were selected based on dG < −20 kcal/mol and iPTM > 0.8. Candidates were subsequently filtered manually by stability of the predicted structures and to exclude sequences with extended charged stretches or alternating charged residues, yielding 10 peptides for synthesis (Figure 2A).

**FIGURE 2:**
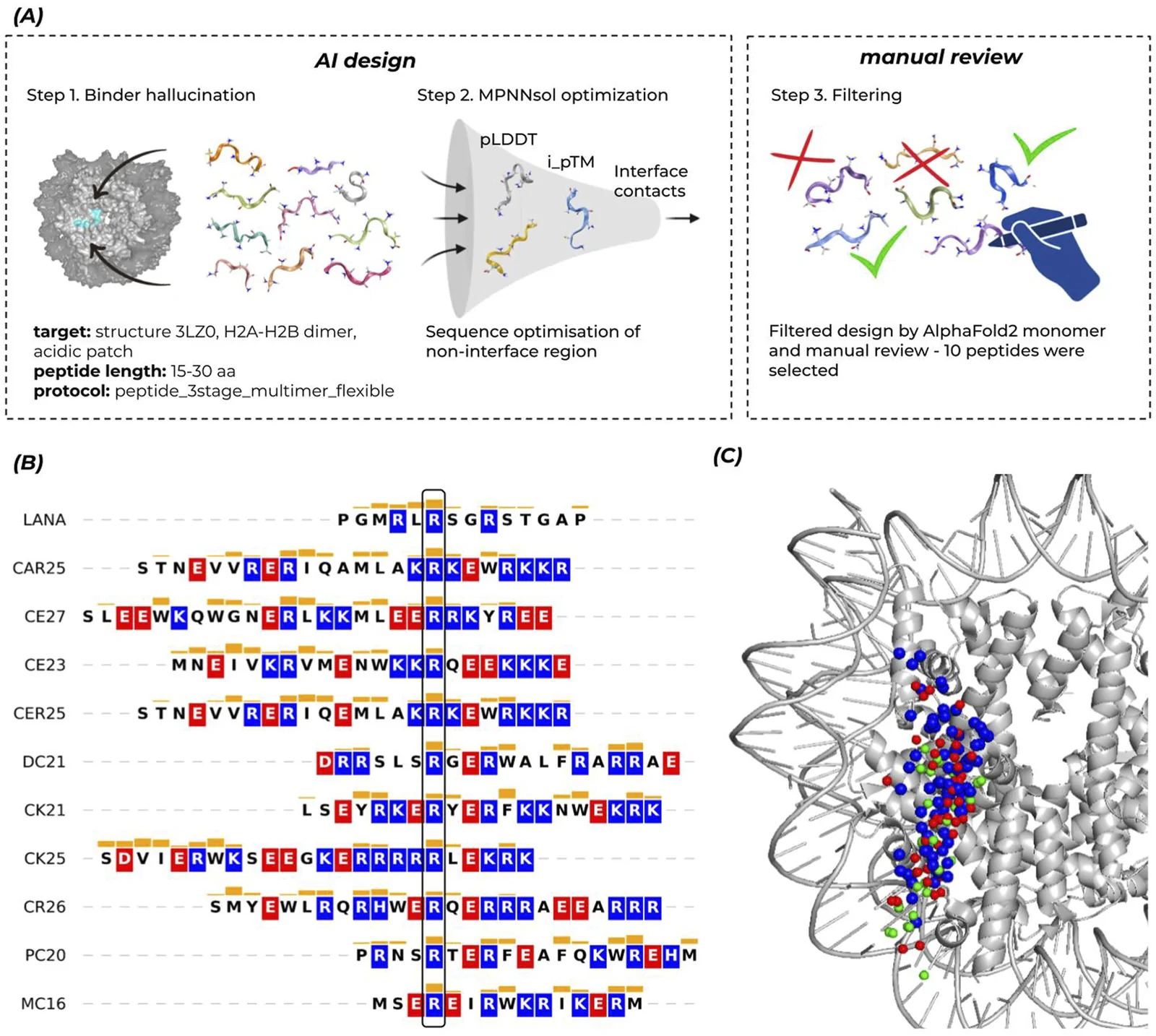
Peptide design and sequence analysis. **(A)** Nucleosome binding peptide design pipeline. Left–*de novo* peptide design using the BindCraft algorithm. Right–manual selection of candidate peptides. **(B)** Sequences of peptides interacting with the target region aligned by the position of the anchor arginine in the center. The orange bars show the relative number of residue–residue contacts. Positive and negative residues are highlighted by blue and red colors, respectively **(C)** location of positively charged (blue), negatively charged (red), and polar (green) residues of nucleosome-binding peptides around the acidic patch.

### 2.2 Computational structure analysis

Structure analysis was performed using the nucleosome structure bound to the LANA1–22 peptide (PDB ID: 1ZLA (Barbera et al., 2006)) extracted from the nucleosome database (NucleosomeDB, http://nucldb.intbio.org). 10 top peptide structures were superimposed with the reference nucleosome structure (PDB ID: 3LZ0) using sequence–structure alignments performed in PyMOL. An arginine anchor - a key structural motif which is present in the majority of known complexes of nucleosomes with other proteins (Barbera et al., 2006) was defined as the peptide arginine residue interacting with acidic patch residues E61, D90, E92 of H2A (we used numbering for canonical H2A of *X. laevis*) and was used as the reference position for alignment of all peptide sequences. The number of residue–residue contacts between peptide and acidic patch residues was calculated using a 4 Å distance cutoff. Sequence alignments, contact analysis, and visualization were performed using custom Python scripts based on Biopython, NumPy, Pandas, and Matplotlib.

### 2.3 Peptide synthesis or production

All peptides were synthesized by Shanghai Royobiotech Co., Ltd. (China). Peptide LANA(1–22) was synthesized with a N-terminal Cy5 fluorescent label for use as a FRET acceptor. Candidate artificial peptides were synthesized without fluorescent labels. Cysteine residues were incorporated into the sequences of the designed peptides to enable potential fluorescent labeling. Lyophilized peptides were dissolved in mQ water; concentrations were determined using a Qubit fluorometer (Thermo Scientific, United States) with the QuDye Protein quantification kit (Lumiprobe, United States) and using the previously published 214 nm absorption protocol (Kuipers and Gruppen, 2007) (Supplementary Material S1.1, Supplementary Figure S4).

### 2.4 Nucleosomal DNA preparation

147 bp Nucleosomal DNA fragments fluorescently labeled at the 35 bp counting from the start of nucleosome positioning sequence were obtained using PCR from pUC57 plasmid containing Widom 603 nucleosome positioning sequence. It was previously shown that 603 sequence based nucleosomes form a canonical stable structure (Armeev et al., 2025) and serves as more natural sequence for nucleosome assembly for transcription studies (Gaykalova et al., 2015). Briefly, DNA fragments (147 bp long) were amplified via PCR from the pUC57 plasmid containing the Widom 603 nucleosomal DNA sequence using the following primers:

Forward: 5′-ATC​AGT​TCG​CGC​GCC​CAC​CTA​CCG​TGT​GAA​GTC​G [Cy3-dT]CACTCGG-3’ (where Cy3-dT is a nucleotide labeled with Cy3);

Reverse: 5′-ATC​CCA​GGG​ACT​TGA​AGT​AAT​AAG​GAC​GGA​GGG​CCt​CTT​TCA​ACA​TC GAT-3’.

A Cleanup Standard kit (Evrogen, Moscow, Russia) was used to purify the amplified DNA fragments. DNA concentration and purity were assessed spectrophotometrically by A260/A280 absorbance ratio. The integrity and homogeneity of the 147 bp fraction were verified by electrophoresis in 2% agarose gels and 4% polyacrylamide gels (buffer containing 44.5 mM Tris, 44.5 mM boric acid, 1 mM EDTA; рН 7.5–8) stained with SYBR Gold. Where required, the target band was excised from the gel and extracted using the same Cleanup Standard kit (Evrogen, Moscow, Russia).

### 2.5 Histone expression, preparation of histone octamers

All four *H. sapiens* canonical histones were overexpressed in *E. coli* expression strains. *E. coli* BL21 (DE3) strain was used for histones H2B, H2A, H3 and H4 production. Each of the histones was overexpressed in the corresponding strain by growing on LB medium at 37 ^°^C, 220 rpm to an optical density of 0.6–0.8 (OD600). Next, IPTG was added to the final concentration of 0.4 mM and bacterial growing continued at 37 ^°^C, 220 rpm, for approximately 4 h. Recombinant histones were purified, and histone octamer was assembled in strict accordance with the protocol described in (Klinker et al., 2014). Briefly, to prepare histone octamers, H2A, H2B, H3, and H4 histones were combined with molar ratio 1.5:1.5:1:1 in denaturing buffer (50 mM Tris pH 8.0, 7 M GuHCl, 10 mM DTT), dialyzed into refolding buffer (50 mM Tris pH 8.0, 2 M NaCl, 1 mM EDTA, 5 mM β-MeEtOH), and purified via gel filtration chromatography. Denaturing SDS-PAGE with 18% gels was performed to analyze protein purity and content at the stages of overexpression and purification of single histones, as well as during the analysis of fractions after assembly and gel filtration of histone octamers. PAGE with 4% gels was used to check the quality of nucleosomes (10 mM HEPES-Na pH 8.0, 0.2 mMEDTA, 10% glycerol). Similarly, 6% gels were used to analyze the specificity of binding of LANA1-22 to nucleosomes.

### 2.6 Nucleosome assembly

Nucleosome core particles were reconstituted by salt gradient dialysis. Mixing 147 bp Widom 603 DNA and histone octamers at a 1:1.15 M ratio. Dialysis was carried out at 4 ^°^C, using buffers containing 10 mM Tris-HCl pH 8.0, 0.2 mM EDTA, 5 mM β-mercaptoethanol, and and subjected to stepwise dialysis against buffers with decreasing NaCl concentrations (2.0, 1.5, 1.0, 0.75, 0.5, 0.25, and 0.01 M) at +4 °C as described in (Gerasimova et al., 2022). If needed, nucleosomes were purified from nonspecific products by preparative electrophoresis in 4% PAAG as described in (Valieva et al., 2016). The quality of assembled nucleosomes was assessed by native PAGE (4% polyacrylamide, 0.5 × TBE, 10 mM HEPES-Na pH 8.0, 0.2 mM EDTA, 10% glycerol) with fluorescence detection of DNA-incorporated labels using an Amersham Typhoon scanner (Cytiva) with excitation at 532 nm and emission at 580 nm (Cy3 signal). Where required, nucleosomes were additionally purified from nonspecific assembly products by preparative native PAGE followed by overnight elution into HEPES-BSA buffer at +4 °C.

### 2.7 Donor FRET quenching assay

Peptide binding to nucleosomes was assessed using a fluorescence quenching via Forster resonance energy transfer. The assay exploited Cy3 incorporated into the nucleosomal DNA as a fluorescence donor and Cy5 conjugated to the LANA peptide as an acceptor. Upon peptide binding to the nucleosome, proximity of the donor and acceptor leads to a decrease in Cy3 donor fluorescence intensity due to resonance energy transfer. Measurements were performed in buffer containing 10 mM Tris-HCl pH 8.0, 200 µM EDTA, 5 mM 2-mercaptoethanol, 10 mM NaCl. All binding reactions were prepared in low-adhesion microcentrifuge tubes (United States Scientific, United States) (with components added sequentially in the following order: binding buffer, peptide, and nucleosomes). In a competitive binding protocol, the sequential incubation of nucleosomes with 400 nM of Cy5-LANA peptide and then with the designed peptides was performed. Upon displacement of labeled peptide with unlabeled, the Cy3 fluorescence is increased. Each concentration point was measured in five technical replicates of 20 µL and transferred to black low-volume non-binding 384-well microplates (Greiner Bio-One, Germany). Fluorescence intensity measurements were performed with a BMG PolarStar Omega plate reader (BMG Labtech, Ortenberg, Germany) with excitation at 544 nm and emission at 590 nm at multiple gain settings (1,000, 1,500, 2000, and 2,500). To account for the direct contribution of Cy5-labeled peptide to the recorded emission signal, the fraction of acceptor fluorescence intensity at each peptide concentration was determined independently in the absence of nucleosomes and subtracted during data analysis.

### 2.8 Data analysis and binding affinity calculations

Direct peptide binding data were processed using a Python script with the following the equation describing the relationship between fluorescence and peptide concentration:

FI=Fmax 1‐p2+Fmid,n+Fmid,f1‐pp+Fmin p2,

where $p=\frac{\left[P\right]}{Kd+\left[P\right]}$

Where *FI* is the donor fluorescence intensity; *F*_*max*_ *, F*_*mid*_ *, F*_*min*_ are the fluorescence intensities of each NCP state (free, singly occupied, and doubly occupied, respectively); *p* is the probability of peptide binding to one nucleosome site; *[P]* is the peptide concentration; and *Kd* is the dissociation constant of the peptide–NCP complex. The MinMaxScaler was used for normalization of the fluorescence signal. The fluorescence intensity in the absence of the peptide was used as a maximum fluorescence intensity.

To assess the affinity of the candidate peptides, the protocol was adapted for a competitive binding approach. This approach is based on the principle that upon further incubation of nucleosome: LANA (1–22) complexes with the studied peptides, the artificial peptide may displace the reference peptide LANA(1–22) from the complex, reducing FRET efficiency and consequently causing an increase in donor fluorescence. The remaining measurement protocol was identical to that described above for direct donor FRET quenching, with the Cheng–Prusoff equation (Cheng and Prusoff, 1973) applied to calculate the dissociation constant in the competitive format:

$${Kd}_{l}=\frac{{IC}_{50}}{1+\frac{\left[LANA\right]}{Kd\left(LANA\right)}}$$

where *Kd*_*l*_ is the dissociation constant of the nucleosome–peptide complex; *IC*_*50*_ is the peptide concentration at which 50% displacement of LANA(1–22)–Cy5 is observed; *[LANA]* is the concentration of LANA(1–22)–Cy5; and *Kd(LANA)* is the dissociation constant of the nucleosome–LANA(1–22)–Cy5 complex. The MinMaxScaler was used for normalization of the fluorescence signal. The fluorescence intensity in the presence of the highest peptide concentration was used as a maximum fluorescence intensity.

### 2.9 Computational prediction of peptides’ binding affinity

Estimation of the peptides affinity via calculation of the Gibbs free energy of binding (dG) using a computational approach was carried out using the FoldX algorithm (Schymkowitz et al., 2005) after applying RepairPDB tool. Complexes of peptides with C and D chains of the 3LZ0 nucleosome structure constructed by the BindCraft algorithm were used. In the case of LANA peptide, using the alphafold2_multimer_v3 model, a complex was constructed based on the sequences of chains C and D from 3LZ0 and the complete LANA(1–22) chain from 1ZLA structure. Default rotamer tables and FoldX parameters were applied to derive the interaction energies.

## 3 Results

### 3.1 Structural predictions and interaction with the acidic patch

Primarily, we focused on a computational analysis of generated and selected peptides. All of the ten peptide candidates have an “anchor” arginine residue which contacts with the E61, D90 and E92 residues of a H2A histone protein forming the pocket (Figure 2B). It was previously shown that this interaction plays a crucial role in peptide binding to the acidic patch of the nucleosome (McGinty and Tan, 2016; Lagadec et al., 2022). Interestingly, despite the absence of the reference peptides during the sequence design, finally all the peptides contained this ‘anchor’ arginine emphasizing the significance of its interaction. Even though peptides present a broad range of different interaction residues (including charged, polar and hydrophobic), the contacts are mostly mediated by charged amino acids, neutralizing the charge of the acidic patch (Figure 2C). It is also should be noted that prediction do not consider the potential peptide-DNA interaction and interaction with other histones in the structure of the NCP requiring a two-fold objective for the experimental validations: first, to verify the binding affinity, and second, to test the specificity of the interactions.

### 3.2 Assay conditions optimization and validation

To assess the binding affinity of peptides the quantitative FRET quenching approach was developed. This assay is based on an increased proximity of the donor and acceptor labels during the peptide binding to the nucleosome core particle, which leads to a decrease in Cy3 donor fluorescence intensity due to resonance energy transfer.

First of all, nucleosomes were assembled with a Cy3 fluorescent label at position 35 bp from the start of the nucleosome positioning sequence (Figures 3A,B). The optimal nucleosome concentration was determined using the Cy3-labeled DNA fragments and the equipment ability to detect fluorescence in the sample in a stable and reproducible manner. The LANA(1–22) peptide was used for these studies, which was synthesized with a fluorescent Cy5 label attached to its N-terminus. In addition, the contribution of the Cy5 label fluorescence to the Cy3 detection channel was measured independently at each peptide concentration and used as a correction factor during data analysis (Figure 3C).

**FIGURE 3:**
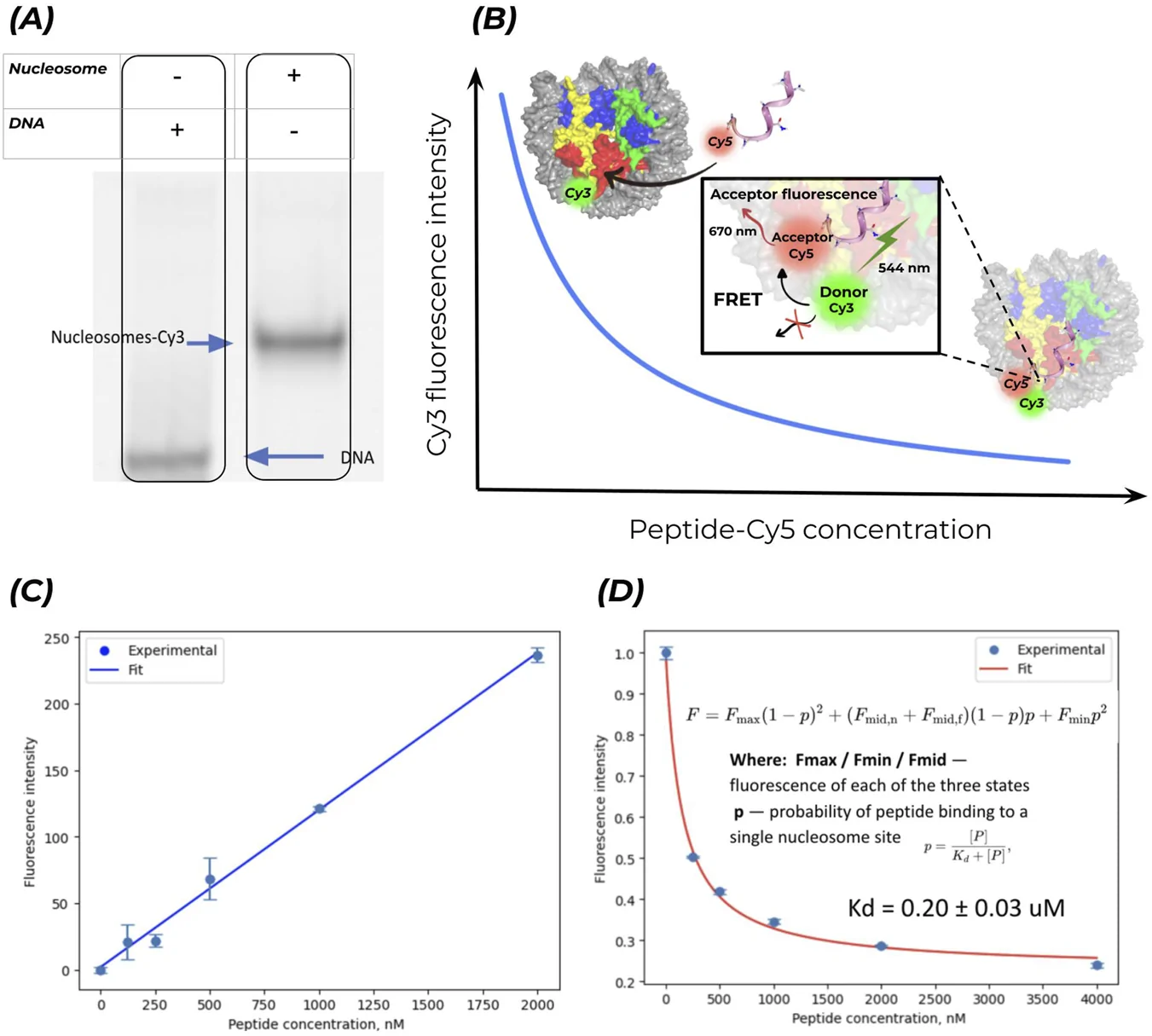
Assay conditions optimization and validation. **(A)** Analysis of the nucleosome assembly via gel electrophoresis. Nucleosomal Cy3-labeled DNA (on the left) and nucleosomes (on the right) were subject to 4% PAGE. The gel was scanned using a Typhoon imager in multiple channels to detect Cy3 fluorescence. **(B)** Schematic representation of donor FRET quenching assay. A Cy5-labeled peptide with high affinity for the acidic patch binds to the nucleosome, resulting in FRET-based quenching of Cy3 fluorescence. **(C)** Dependence of Cy5 fluorescence intensity on the concentration of LANA1-22. Fluorescence intensity increases linearly with concentration, allowing correction for Cy5 spectral cross-talk in the Cy3 channel. **(D)** Dependence of Cy3 fluorescence intensity on the concentration of LANA1-22 with correction for Cy5 contribution to the Cy3 channel.

In order to demonstrate the feasibility of the proposed method we determined the binding affinity of previously known complex of the nucleosome with the peptide LANA(1–22). The dissociation constant of the nucleosome–LANA(1–22) complex was determined to be 0.2 ± 0.03 µM, which is consistent with previously published values obtained by fluorescence polarization and microscale thermophoresis (Corbeski et al., 2024) (Figure 3D). It should be noted that differences in Kd values may be attributable to the charge of the fluorescent label attached to the N-terminus of the peptide. In fluorescence polarization experiments, the FAM label at the N-terminus of LANA(1–22) carries a weak negative charge at the pH values used (Sjöback et al., 1995), which may reduce the affinity of the peptide and consequently increase the dissociation constant. Conversely, in the donor FRET quenching assay, the Cy5 label attached to the peptide terminus bears no charge or a slight positive charge under identical pH conditions (Mujumdar et al., 1993), thus not affecting or potentially enhancing the electrostatic interactions of the peptide with the nucleosome acidic patch.

### 3.3 Experimental validation of designed peptide binding

Dissociation constants of the artificial peptide candidates with NCPs were measured using the competitive binding variant of the optimized donor FRET quenching assay (Figure 4A). Among the selected sequences, two peptides exhibited high binding affinity with submicromolar dissociation constants (Figure 4B), three peptides showed affinity in the 1–5 µM range, and the remaining peptides did not display detectable competitive displacement of LANA(1–22)–Cy5 (Table 1). It should be also emphasized that the competitive binding protocol specifically measures binding of the peptide to the acidic patch of the NCP, as the assay is based on displacement of LANA(1–22)–Cy5, which is known to bind specifically at this site (Supplementary Material S1.2, Supplementary Figures S5.1–5.3).

**FIGURE 4:**
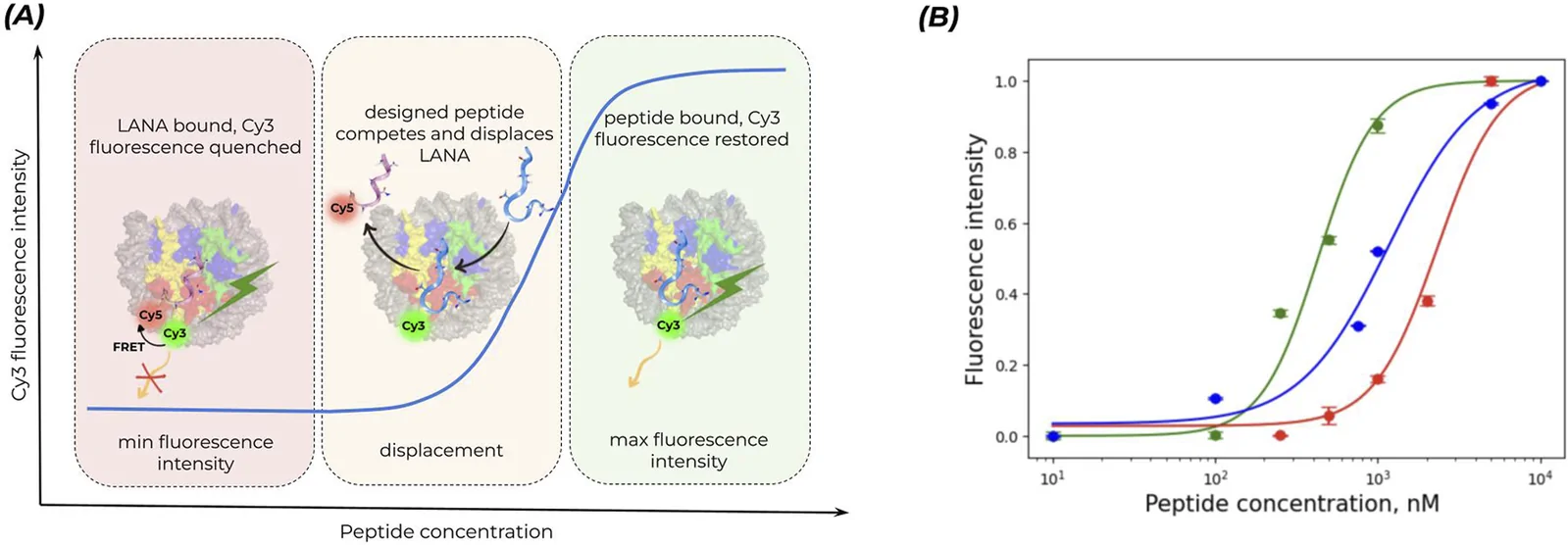
Experimental validation of designed peptide binding. **(A)** Schematic representation of competitive binding quantitative FRET quenching assay. Cy5-labeled peptide binding to the nucleosome results in minimal Cy3 fluorescence intensity due to FRET quenching. The designed peptide with high affinity for the acidic patch displaces Cy5-labeled peptide, leading to restoration of Cy3 fluorescence. **(B)** Results of measurements of the dissociation constants for complexes of NCPs with artificial peptides CK25 (red), CK21 (blue), DC21 (green) exhibiting the highest affinity for the acidic patch of the nucleosome (the dots represent fluorescence intensity values ± SEM).

**TABLE 1:** Sequences, predicted and experimentally estimated NCP-peptde Kd values.

Name	Sequence	Estimated Kd value, uM	Predicted dG (FoldX), kcal/mol
**LANA(1–22)**	MAPPGMRLRSGRSTGAPLTRGS	0.2 ± 0.03	−23.51
**DC21**	DRRSLSRGERWALFRARRAEC	0.22 ± 0.05	−22.06
**CK21**	CLSEYRKERYERFKKNWEKRK	0.58 ± 0.07	−23.41
**CK25**	CSDVIERWKSEEGKERRRRRLEKRK	1.16 ± 0.17	−28.28
**CER25**	CSTNEVVRERIQEMLAKRKEWRKKR	≈2	−23.57
**CAR25**	CSTNEVVRERIQAMLAKRKEWRKKR	≈2	−21.8
**CE27**	CSLEEWKQWGNERLKKMLEERRKYREE	-	−21.47
**CE23**	CMNEIVKRVMENWKKRQEEKKKE	-	−16.67
**PC20**	PRNSRTERFEAFQKWREHMC	-	−19.31
**MC16**	MSEREIRWKRIKERMC	-	−20.49
**CR26**	CSMYEWLRQRHWERQERRRAEEARRR	-	−18.27

It has previously been shown that peptides with high affinity for the acidic patch of nucleosome core particles - such as peptides derived from the natural proteins LANA and CENP-C (LANA(1–22) and CENP-Cmotif) - have a stabilizing effect on the structure of nucleosome core particles (Ali-Ahmad et al., 2019; Oleinikov et al., 2023), which may consist of both a reduction in the spontaneous unwinding of the nucleosomal DNA fragment from the histone octamer and more global rearrangements affecting the nucleosome core particle octamer itself. Based on the presented results, and given the high affinity of some of the selected peptides, it can be assumed that synthetic peptides are also capable of exerting a stabilizing effect on the structure of nucleosomal core particles; however, this should be confirmed experimentally.

## 4 Discussion

In this study we demonstrated that an AI-guided BindCraft pipeline can be successfully used for nucleosome-binding peptide design. About a hundred peptide sequences with a broad range of affinity predictions were generated using the BindCraft algorithm, the best forty of which were selected for a manual filtering according to the charged amino acid residue localization in the generated sequences. Ten peptide candidates with highest predicted affinity finally were experimentally validated. All of the structures generated are alpha-helical. This fact is mediated by the AlphaFold2 structure scoring part of the BindCraft algorithm which prefers the alpha-helical structures due to its better predictability (McDonald et al., 2023; Pacesa et al., 2025). Notably, none of the peptides obtained resemble their natural counterparts in terms of either structure or sequence (Supplementary Material S1.3, Supplementary Figure S7); however, they exhibit similar interaction patterns, particularly with the pocket region of the anchor arginine (Oleinikov et al., 2023).

We also developed a protocol for nucleosome binding peptide affinity measurements in two variants: one is the straight quantitative donor FRET quenching approach and the competitive binding protocol for unlabeled peptides. Such an approach allowed us both to estimate the dissociation constant of the complex of previously known natural peptide LANA(1–22) with the nucleosome core particles and to evaluate the binding affinity of generated and filtered peptide candidates. This approach requires comparatively low concentrations of nucleosomes (10 nM in our protocol) and bulk FRET measurements while reducing the contribution of high concentrations of Cy5-labeled peptide through the detection of donor label fluorescence, and eliminating the need for complex single-molecule assays. The method can also be considered more advantageous compared to gel shift (EMSA)-based approaches (Novikov et al., 2020). In the latter, due to the electrostatic nature of the interactions between the peptides and the nucleosome acidic patch, the peptide may dissociate from the complex during electrophoresis, altering its effective concentration within the band and thereby complicating accurate Kd determination. Moreover, competitive variant of the method indicate the highly specific binding of the peptides due to the high affinity for the acidic patch of the nucleosome of reference natural peptide.

Half of the manually selected peptides did not demonstrate the ability to replace LANA(1–22) peptide in the binding site on the nucleosome surface. However, three peptides had a micromolar binding affinity and two more peptides (DC21 and CK21) demonstrated the submicromolar Kd values in a complex with the nucleosome particles. Our proof-of-concept study identified a peptide with an affinity comparable to that of a peptide derived from a natural protein using only a limited number of experimentally tested candidates, highlighting the potential of modern AI-powered protein design algorithms to reduce the extent of high-throughput experimental screening.

At the same time computational analysis of the binding affinity provides interesting insights into modern tools of computational interaction energy scoring such as FoldX. Although the ranking of the peptides according to the predicted dG score differed from the ranking according to the experimental measurements, all of the peptides that demonstrated a high value of the experimentally measured affinity for the acidic patch of the nucleosome also had a lower than −21.5 kcal/mol computationally predicted dG score, while the non-binding peptides demonstrated higher scores. Moreover, even though the LANA(1–22) peptide exhibited a dG score comparable to the calculated scores of the other peptides, its experimentally measured Kd value was the lowest. Thus, while on the one hand, computational interaction energy estimations provide an opportunity to filter the designed candidates, on the other hand, the experimental validation is still a requirement.

In the context of therapeutic applications, developments in the field of peptide modulators which occupy a niche between small molecules and biological macromolecules, combining the advantages of both groups (Zheng et al., 2025), have gained renewed relevance in recent years. Nucleosome-binding peptides have been studied as antiviral (Beauchemin et al., 2014), antitumor (Davey et al., 2017) agents and have a high potential to be used for directed genome functioning regulation. For instance, it has previously been shown that peptides with high affinity for the nucleosome acidic patch, such as LANA(1–22) and CENP-C motif (Ali-Ahmad et al., 2019; Oleinikov et al., 2023), exert a stabilizing effect on NCP structure (Kniazeva et al., 2022). However, previously established rational design of nucleosome binding GMIP1 peptide based on RCC1 (Teles et al., 2020) fragment with dual affinity for the acidic patch and the DNA exhibited the controversial effects of GMIP1 such as nucleosome precipitation, nonspecific DNA interactions and no effect on chromatin compaction (Teles et al., 2020). Here we proposed a competitive assay, which implies specific acidic patch binding. Thus, the specific peptide binding affinity and absence of nonspecific peptide-DNA interactions serve as an important property affecting potential practical applications of nucleosome-binding peptides.

Based on the high affinity of the top candidates identified here, it can be hypothesized that the artificial peptides similarly to natural peptides are capable of stabilizing nucleosomes, although this remains to be confirmed experimentally. Furthermore, given that nucleosomal DNA dynamics substantially influence the conformational dynamics of chromatin fibers (Armeev et al., 2021), such peptides may be considered as potential modulators not only of individual nucleosomes but also of higher-order chromatin structures (Armeev et al., 2019). Together with additional sequence elements targeting the chromatin modulating peptide to the specific compartment in the nucleus which can be designed using ProtGPS (Kilgore et al., 2025), such peptide serves as an efficient agent for manipulations with the genome 3D-organization.

Nucleosome binding peptides may be also considered as auxiliary components for future therapeutic modalities. Such peptides may be used as targeting and binding elements for epigenetic engineering instrumentation. Thus, the CRISPR-Chrom platform uses the Cas9 protein fused with chromatin-binding peptides in order to enhance the ability of the Cas9 to bind even in nucleosome-occluded genomic regions (Ding et al., 2019). It was also previously shown that linking acidic patches of two nucleosomes leads to “chromatin arrest” mediating the cytotoxicity of such compounds (Davey et al., 2017). In this case, nucleosome-binding peptides with high affinity for the acidic patch may become ‘building blocks’ for the creation of similar tools with a high potential for anticancer therapeutic implications (Shaytan et al., 2022; Kovalev et al., 2025)).

In conclusion, this study demonstrates a high experimental validation success rate for AI-designed nucleosome-binding peptides, yielding binders with affinities comparable to those of natural sequences despite relying on only a limited experimental screening effort. Together with the experimental assay developed here, this work establishes a versatile platform for applications ranging from peptide-based therapeutics to the development of advanced genetic and epigenetic editing tools.

## Data Availability

The original contributions presented in the study are publicly available. This data can be found here: https://doi.org/10.5281/zenodo.23265003.
