# Supplementary material for: Design and validation of nucleosome binding peptides using BindCraft
Source: Front Bioeng Biotechnol. 2026 Oct 22;14:1946220. doi: 10.3389/fbioe.2026.1946220 (PMC13736603; doi:10.3389/fbioe.2026.1946220)
Supplement: Supplementary file 1 [file DataSheet1.pdf]

Supplementary Material

1 Supplementary Methods

1.1 Peptide concentration measurements.

Additional peptide concentration verification was carried on in accordance with (Kuipers and Gruppen, 2007). Peptides were subjected to absorbance measurements using the Cary 100 Scan spectrophotometer (Varian Inc., USA) in a buffer containing 20% (v/v) acetonitrile and 0.1% (v/v) formic acid (~pH 3). Absorbance at a wavelength of 214 nm was used as the measure of the result. (see Supplementary Figure 4)

1.2 Peptide - DNA interaction analysis

Measurements were performed in a buffer containing 10 mM Tris-HCl pH 8.0, 200 µM EDTA, 5 mM 2-mercaptoethanol, 10 mM NaCl. Each sample containing 10nM Cy3-labeled nucleosomal DNA fragments and different concentrations of peptide were prepared in low-adhesion microcentrifuge tubes (USA Scientific, USA). Each concentration point was measured in five technical replicates of 20 µl and transferred to black low-volume non-binding 384-well microplates (Greiner Bio-One, Germany). Fluorescence intensity measurements were performed with a BMG PolarStar Omega plate reader (BMG Labtech, Ortenberg, Germany) with excitation at 544 nm and emission at 590 nm at multiple gain settings (1000, 1500, 2000, and 2500). (see Supplementary Figures 5.1-5.3)

1.3 Comparison of designed peptides with known nucleosome acidic-patch-binding sequences

The initial structural dataset was obtained from NucleosomeDB using the previously described procedure for identification and structural alignment of nucleosome-containing PDB entries (https://nucldb.intbio.org/, Last update 27.03.2026). The resulting dataset was screened for structures containing non-histone proteins or peptides contacting the nucleosomal acidic patch. The acidic-patch region was defined using PDB structure 1ZLA as the structural reference. Structures containing at least one interaction with this region were further examined for the presence of an arginine side chain inserted into the acidic-patch pocket. An arginine was considered to occupy the pocket when its atoms were located within 5 Å of the reference acidic-patch region. This distance-based selection follows the general strategy previously used to identify arginine-mediated acidic-patch interactions (Oleinikov et al., 2023).

For each selected structure, the sequence of the interacting protein around the acidic-patch-binding arginine (anchor arginine) was extracted and mapped onto a common sequence coordinate system centered on this residue. Residues not resolved in the experimental structure were represented as gaps. The resulting sequences were clustered according to their mutually compatible resolved residues. Pairwise distances were defined as the number of positions at which both structures contained a resolved residue but the amino-acid identities differed; positions unresolved in either structure were ignored. Agglomerative clustering with complete linkage was then used to group sequences with no conflicting resolved residues. For visualization, each cluster was

represented by the member containing the largest number of resolved residues, while all PDB entries compatible with that representative sequence were retained and reported.

Designed peptides were aligned to the structural sequences using the designated anchor arginine as a fixed reference position. Each structural sequence was compared with all designed peptides using the BLOSUM62 substitution matrix. Similarity was calculated over positions resolved in the experimental structure and normalized to the maximum BLOSUM62 self-similarity possible for those positions; negative substitution scores were treated as zero. In parallel, structural coverage was reported as the number of resolved residues overlapping the designed peptide relative to the peptide length. Each structural cluster was assigned to the designed peptide with the highest sequence similarity, with coverage used to resolve ties. Designed-peptide groups were ordered by the number of associated PDB entries, and structures within each group were ordered primarily by the number of resolved residues, followed by sequence similarity [Supplementary Figure 7].

2 Supplementary Figures and Tables

2.1 Supplementary Figures

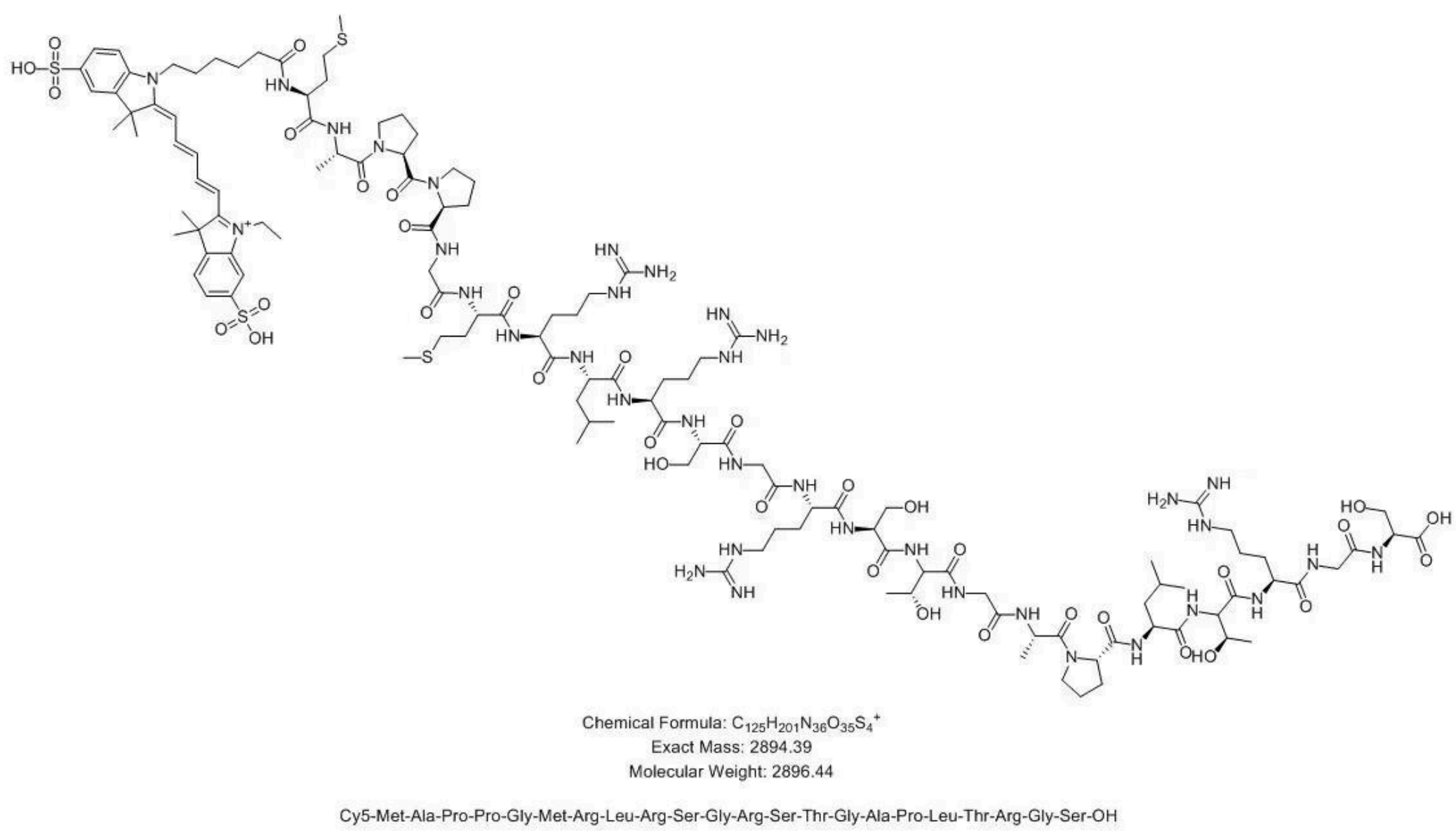

Supplementary Figure 1. ChemDraw Structure of Cy5-LANA(1-22) peptide

Flow rate: 1.0ml/min

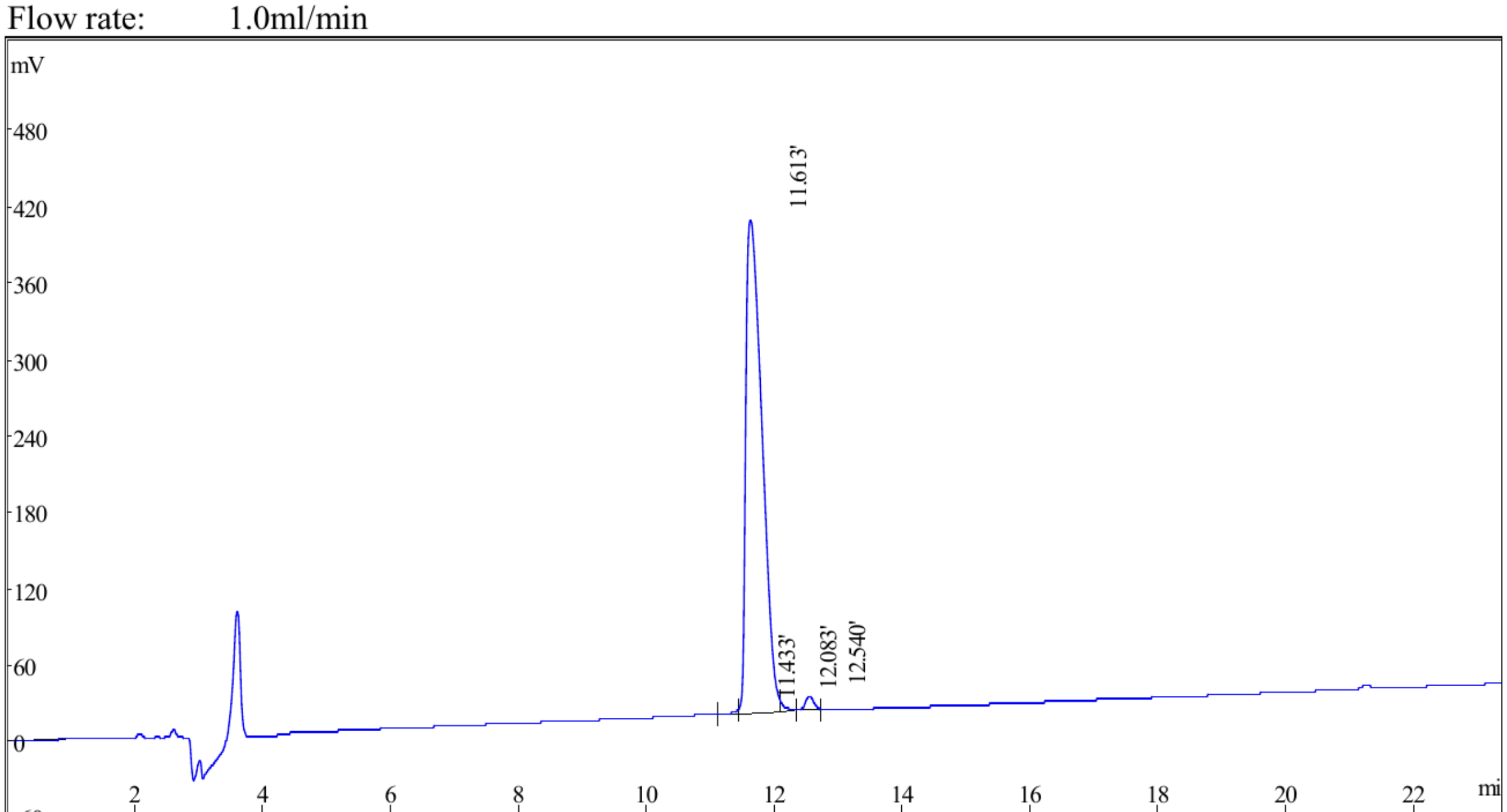

Rank	Time	Conc.	Area	Height
1	11.433	0.1566	10456	3160
2	11.613	97.8429	6532048	386182
3	12.083	0.5995	40026	7509
4	12.540	1.4010	93535	10413
Total		100	6676065	407264

Supplementary Figure 2.1. HPLC analysis of Cy5-LANA(1-22) peptide. Gemini-NX 5μ C18 110A, 4.6*250mm column, Solvent A: 0.1% Trifluoroacetic Acid in 100% Acetonitrile, Solvent B: 0.1% Trifluoroacetic Acid in 100% Water, wavelength: 220nm.

Flow rate: 1.0ml/min

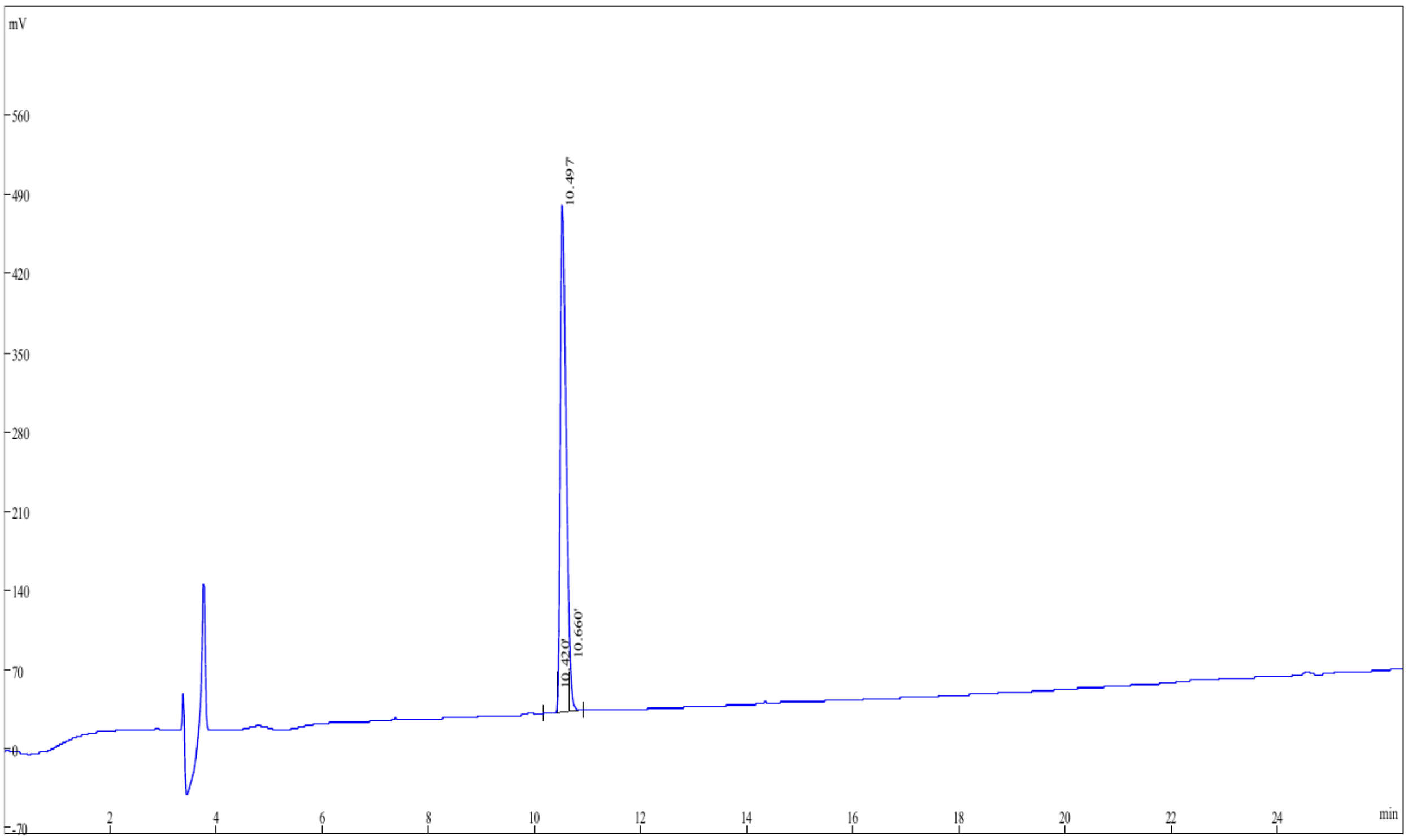

Rank	Time	Area	Conc.
1	10.420	15124	0.4374
2	10.497	3383164	97.84
3	10.660	59548	1.722
Total			100

Supplementary Figure 2.2. HPLC analysis of CK25 peptide. COSMOSIL Packed Column, 4.6*250mm, 5μm, Solvent A: 0.1% Trifluoroacetic Acid in 100% Acetonitrile, Solvent B: 0.1% Trifluoroacetic Acid in 100% Water, wavelength: 220nm.

Flow rate: 1.0ml/min

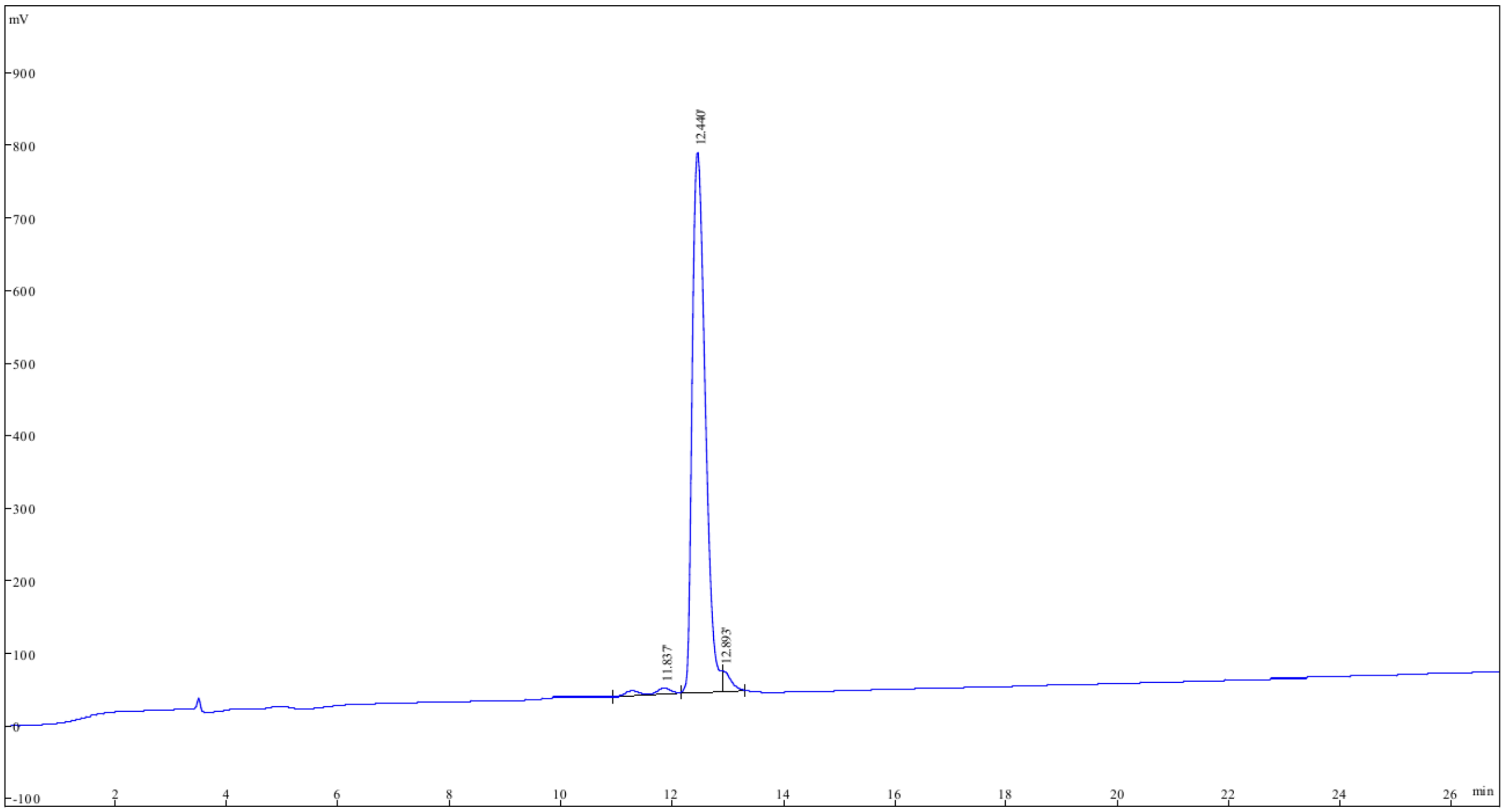

Rank	Time	Area	Conc.
1	11.837	291338	2.28
2	12.440	12195838	95.46
3	12.893	288443	2.258
Total			100

Supplementary Figure 2.3. HPLC analysis of CE27 peptide. COSMOSIL Packed Column, 4.6*250mm, 5µm, Solvent A: 0.1% Trifluoroacetic Acid in 100% Acetonitrile, Solvent B: 0.1%

Trifluoroacetic Acid in 100% Water, wavelength: 220nm

Flow rate: 1.0ml/min

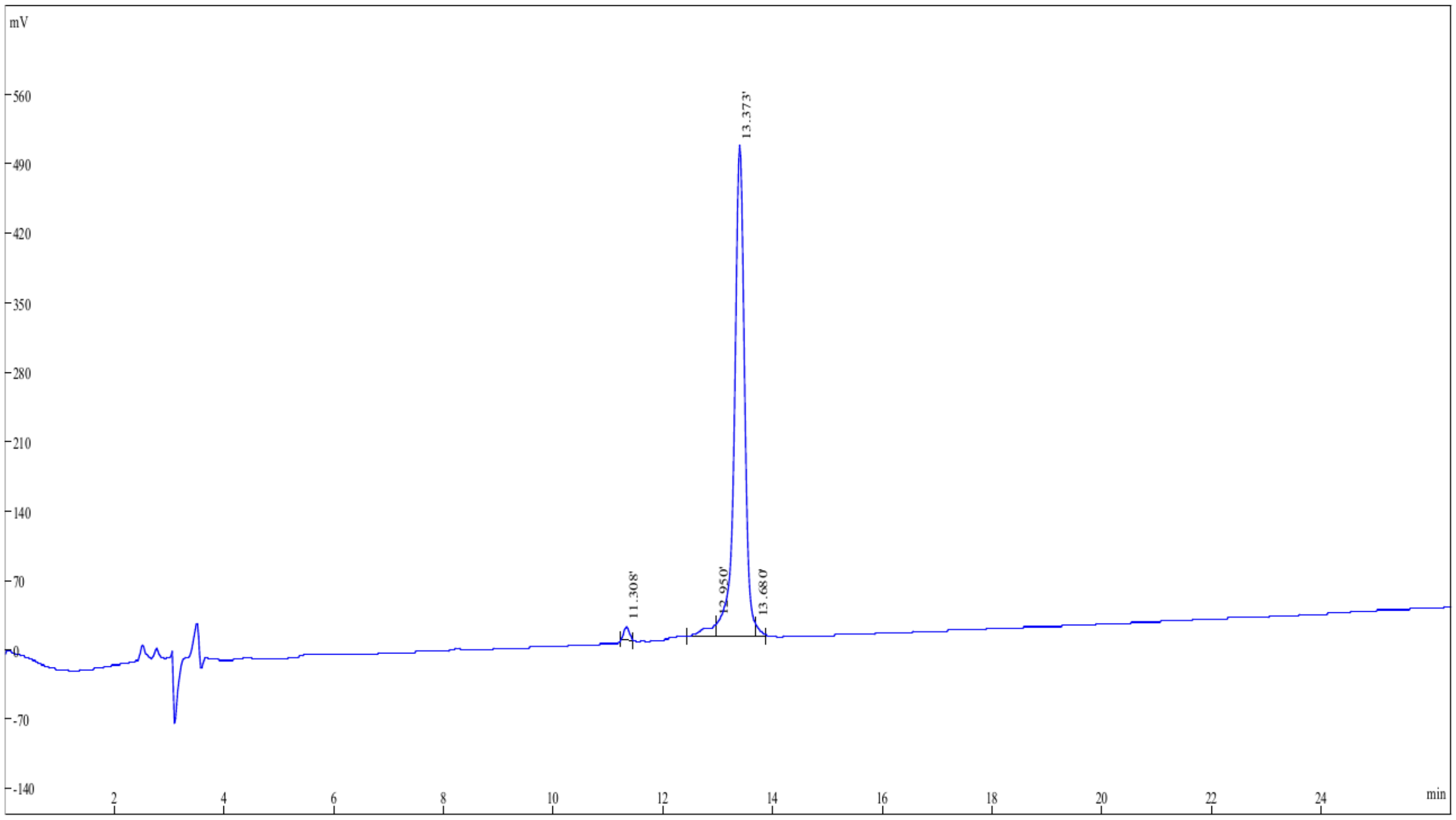

Rank	Time	Area	Conc.
1	11.308	83904	1.273
2	12.950	166916	2.533
3	13.373	6293458	95.49
4	13.680	46415	0.7042
Total			100

Supplementary Figure 2.4. HPLC analysis of CE23 peptide. COSMOSIL Packed Column, 4.6*250mm, 5µm, Solvent A: 0.1% Trifluoroacetic Acid in 100% Acetonitrile, Solvent B: 0.1% Trifluoroacetic Acid in 100% Water, wavelength: 220nm.

Flow rate: 1.0ml/min

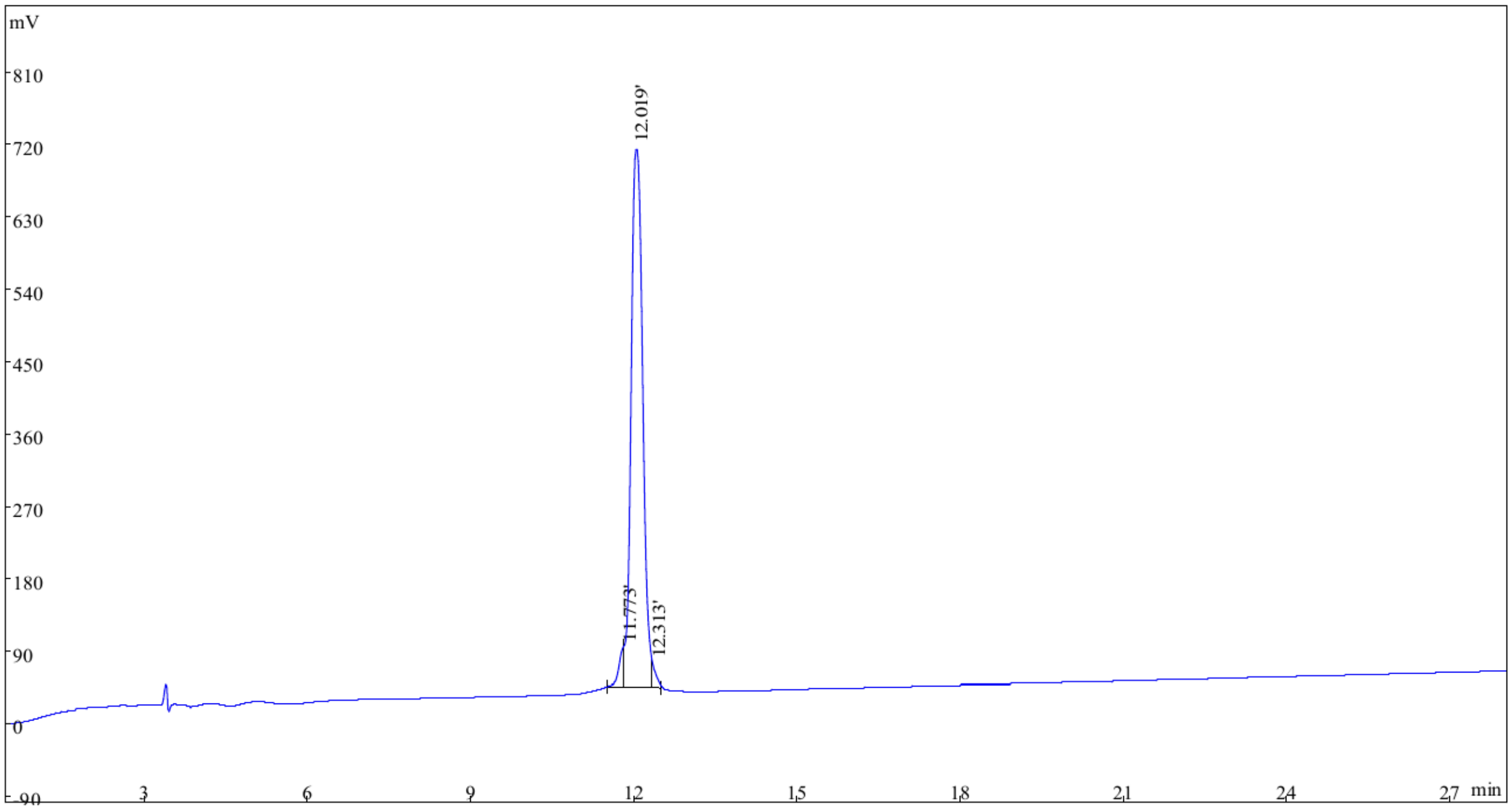

Rank	Time	Area	Conc.
1	11.773	337743	3.541
2	12.019	9079133	95.18
3	12.313	121672	1.276
Total			100

Supplementary Figure 2.5. HPLC analysis of PC20 peptide. COSMOSIL Packed Column, 4.6*250mm, 5μm, Solvent A: 0.1% Trifluoroacetic Acid in 100% Acetonitrile, Solvent B: 0.1% Trifluoroacetic Acid in 100% Water, wavelength: 220nm.

Flow rate: 1.0ml/min

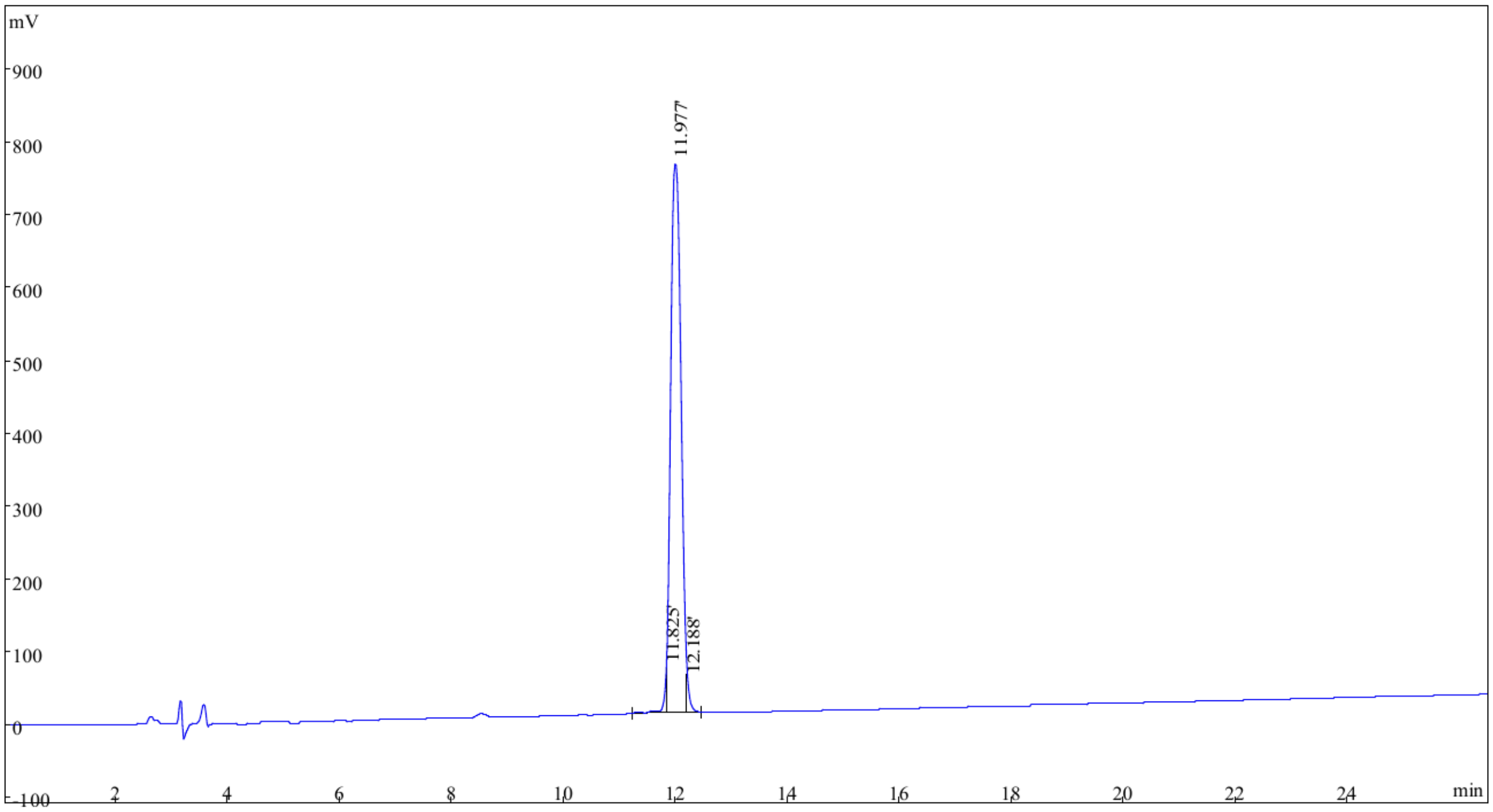

Rank	Time	Area	Conc.
1	11.825	163899	1.796
2	11.977	8842490	96.91
3	12.188	118411	1.298
Total			100

Supplementary Figure 2.6. HPLC analysis of CK21 peptide. COSMOSIL Packed Column, 4.6*250mm, 5µm, Solvent A: 0.1% Trifluoroacetic Acid in 100% Acetonitrile, Solvent B: 0.1% Trifluoroacetic Acid in 100% Water, wavelength: 220nm.

Flow rate: 1.0ml/min

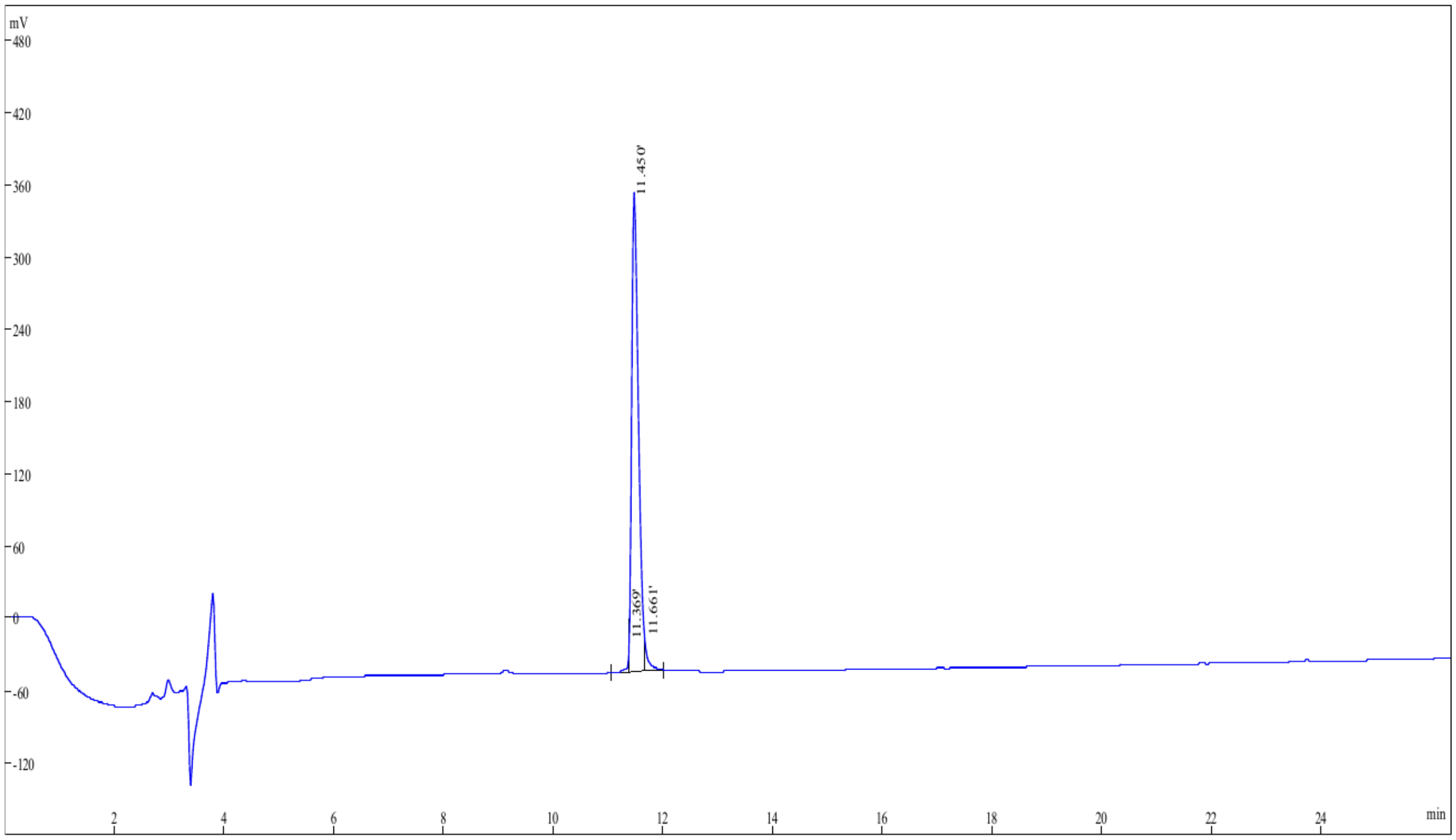

Rank	Time	Area	Conc.
1	11.369	45561	1.296
2	11.450	3386718	96.32
3	11.661	83955	2.388
Total			100

Supplementary Figure 2.7. HPLC analysis of DC21 peptide. COSMOSIL Packed Column, 4.6*250mm, 5µm, Solvent A: 0.1% Trifluoroacetic Acid in 100% Acetonitrile, Solvent B: 0.1% Trifluoroacetic Acid in 100% Water, wavelength: 220nm.

Flow rate: 1.0ml/min

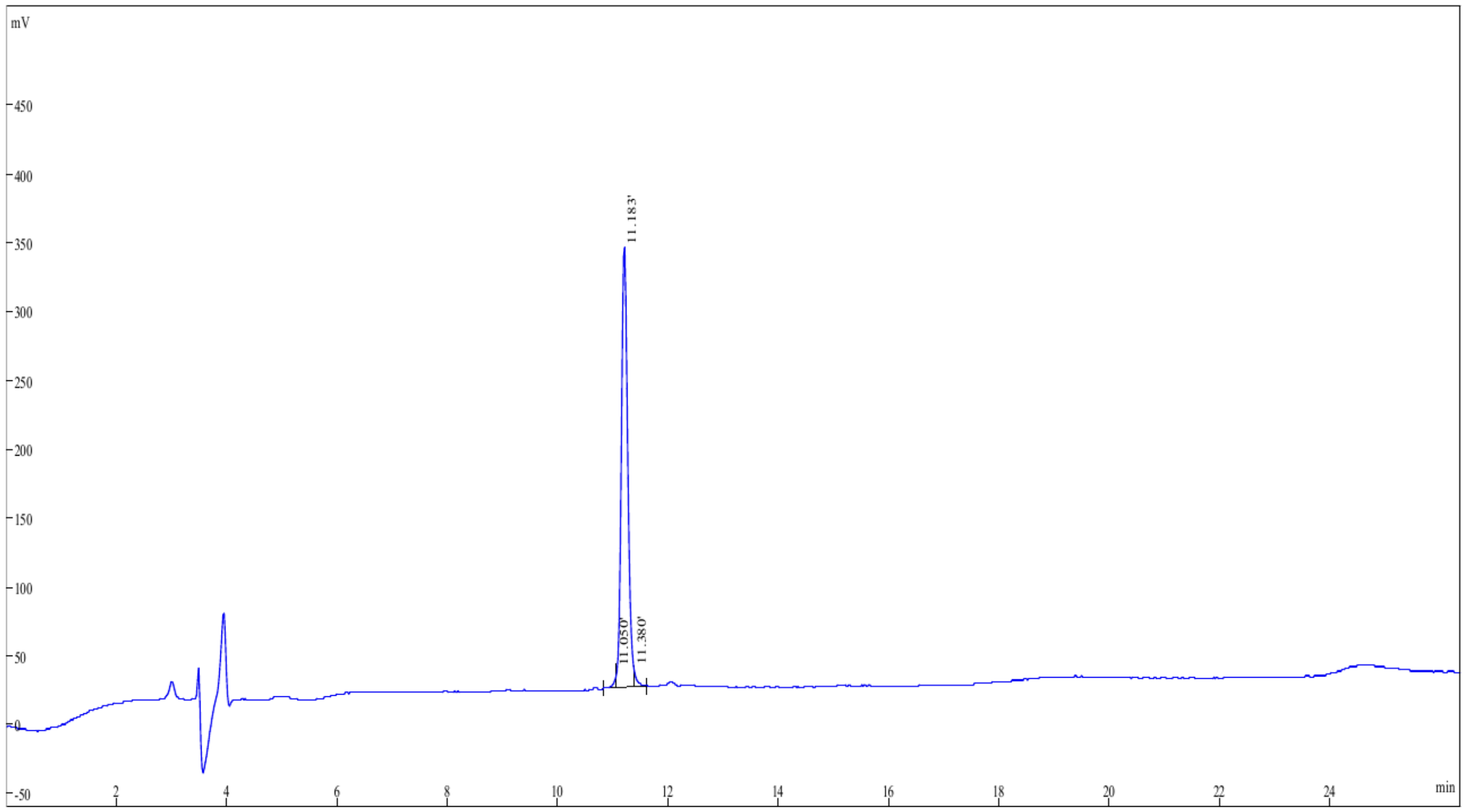

Rank	Time	Area	Conc.
1	11.050	32830	1.244
2	11.183	2575241	97.61
3	11.380	30312	1.149
Total			100

Supplementary Figure 2.8. HPLC analysis of CER25 peptide. COSMOSIL Packed Column, 4.6*250mm, 5µm, Solvent A: 0.1% Trifluoroacetic Acid in 100% Acetonitrile, Solvent B: 0.1% Trifluoroacetic Acid in 100% Water, wavelength: 220nm.

Flow rate: 1.0ml/min

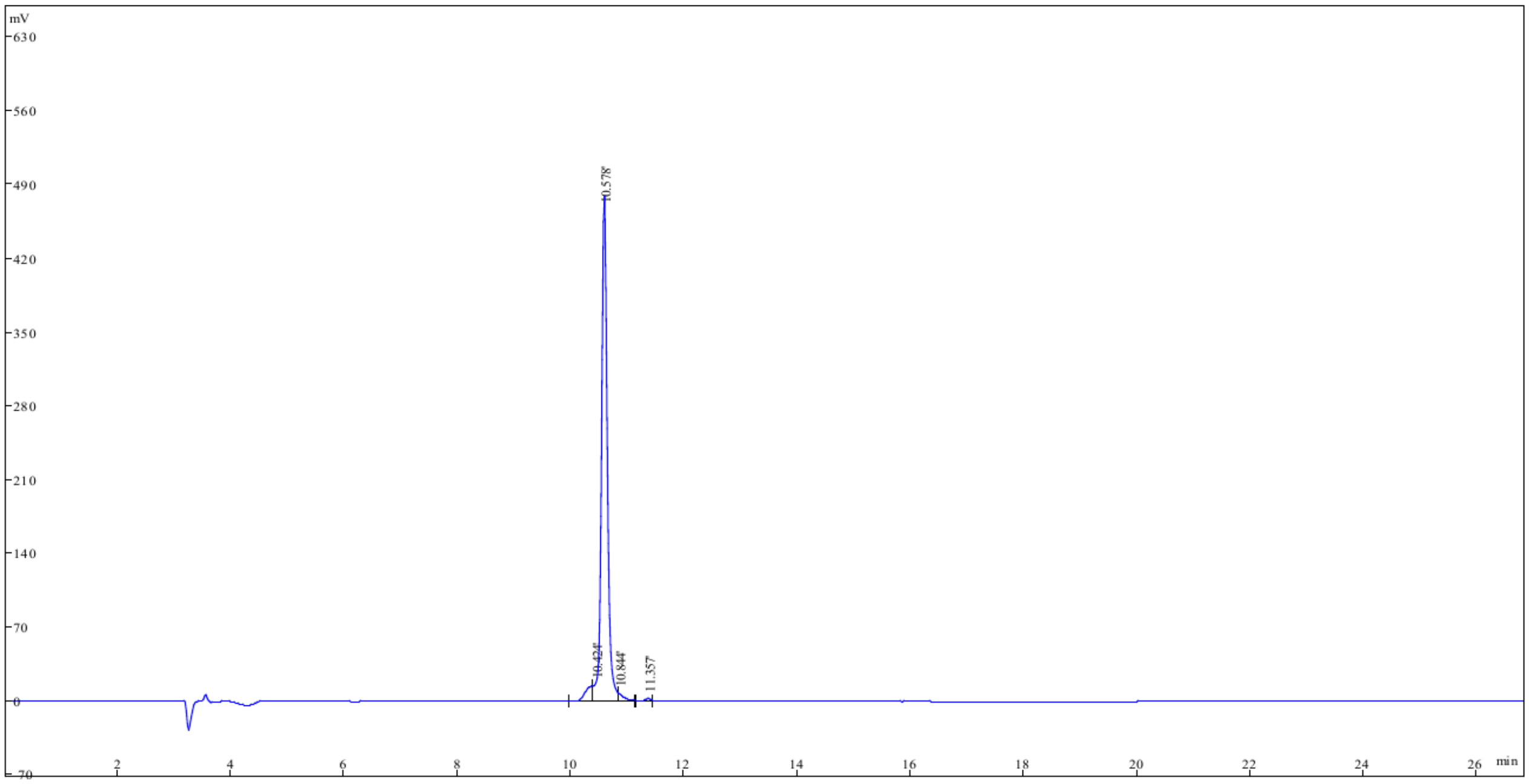

Rank	Time	Area	Conc.
1	10.424	130222	3.655
2	10.578	3391350	95.19
3	10.844	33631	0.9439
4	11.357	7567	0.2124
Total			100

Supplementary Figure 2.9. HPLC analysis of CAR25 peptide. COSMOSIL Packed Column, 4.6*250mm, 5µm, Solvent A: 0.1% Trifluoroacetic Acid in 100% Acetonitrile, Solvent B: 0.1% Trifluoroacetic Acid in 100% Water, wavelength: 220nm.

Flow rate: 1.0ml/min

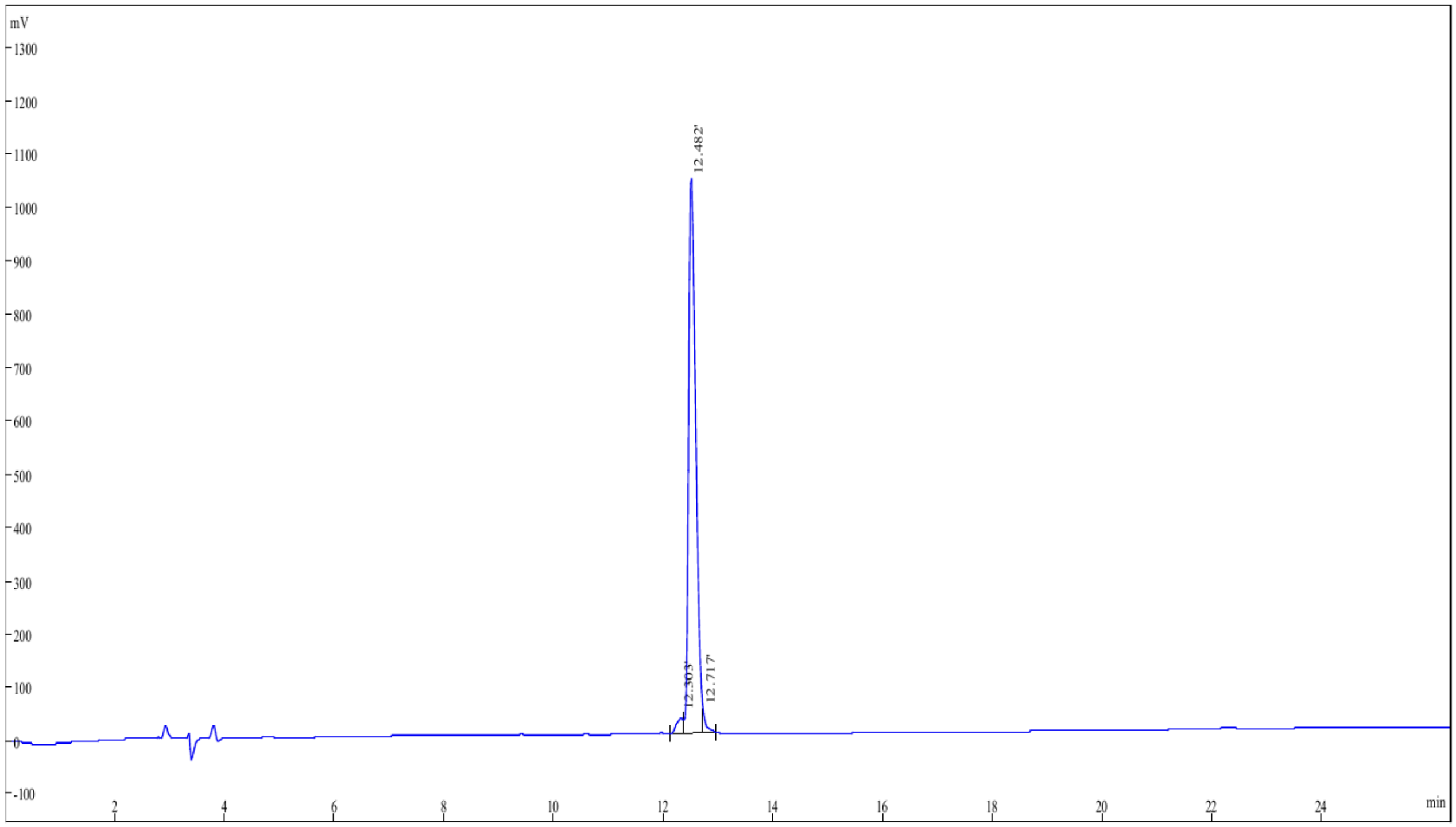

Rank	Time	Area	Conc.
1	12.303	253440	2.569
2	12.482	9509405	96.38
3	12.717	103281	1.047
Total			100

Supplementary Figure 2.10. HPLC analysis of MC16 peptide. COSMOSIL Packed Column, 4.6*250mm, 5µm, Solvent A: 0.1% Trifluoroacetic Acid in 100% Acetonitrile, Solvent B: 0.1% Trifluoroacetic Acid in 100% Water, wavelength: 220nm.

Flow rate: 1.0ml/min

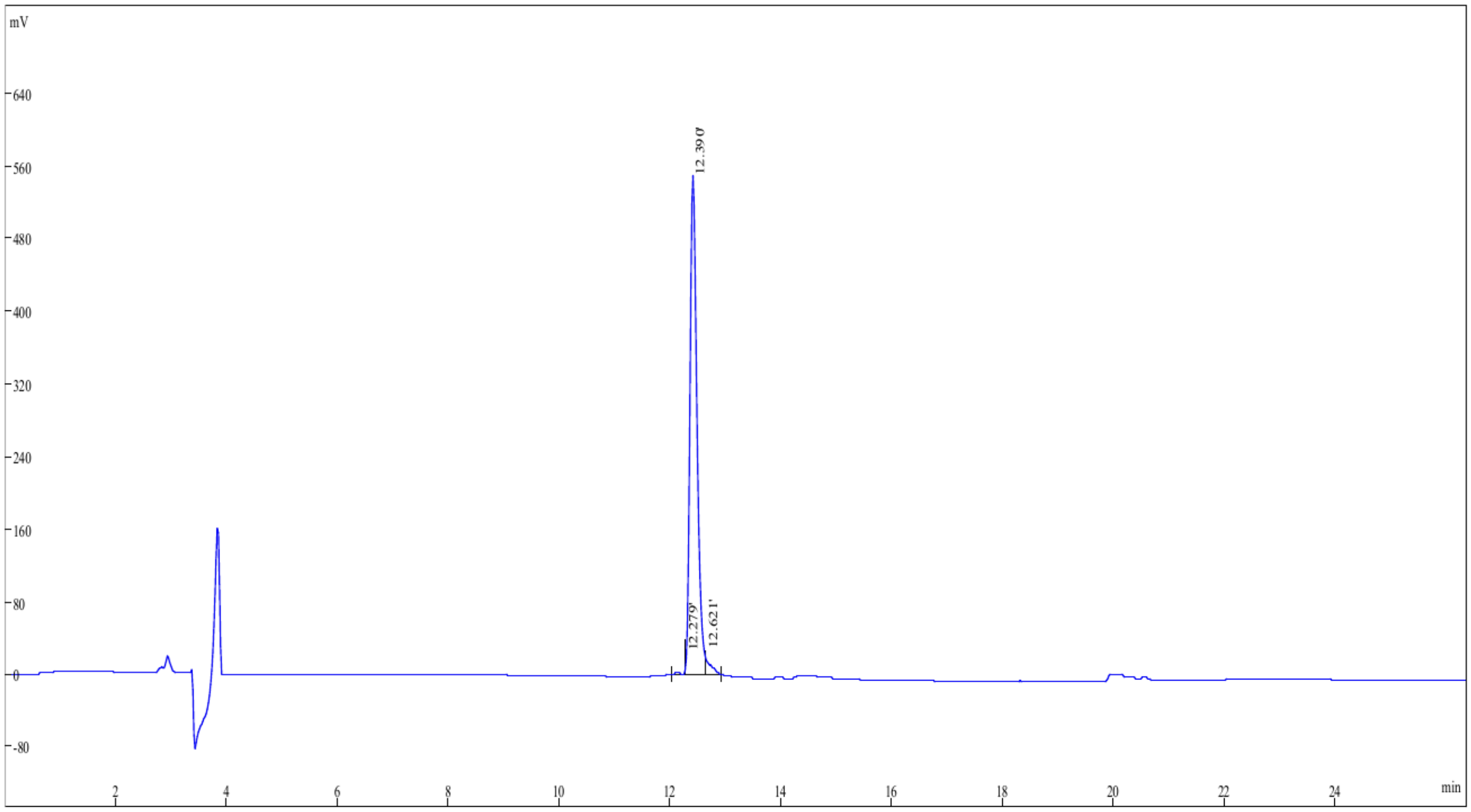

Rank	Time	Area	Conc.
1	12.279	52972	1.059
2	12.390	4818830	96.34
3	12.621	130066	2.6
Total			100

Supplementary Figure 2.11. HPLC analysis of CR26 peptide. COSMOSIL Packed Column, 4.6*250mm, 5µm, Solvent A: 0.1% Trifluoroacetic Acid in 100% Acetonitrile, Solvent B: 0.1% Trifluoroacetic Acid in 100% Water, wavelength: 220nm.

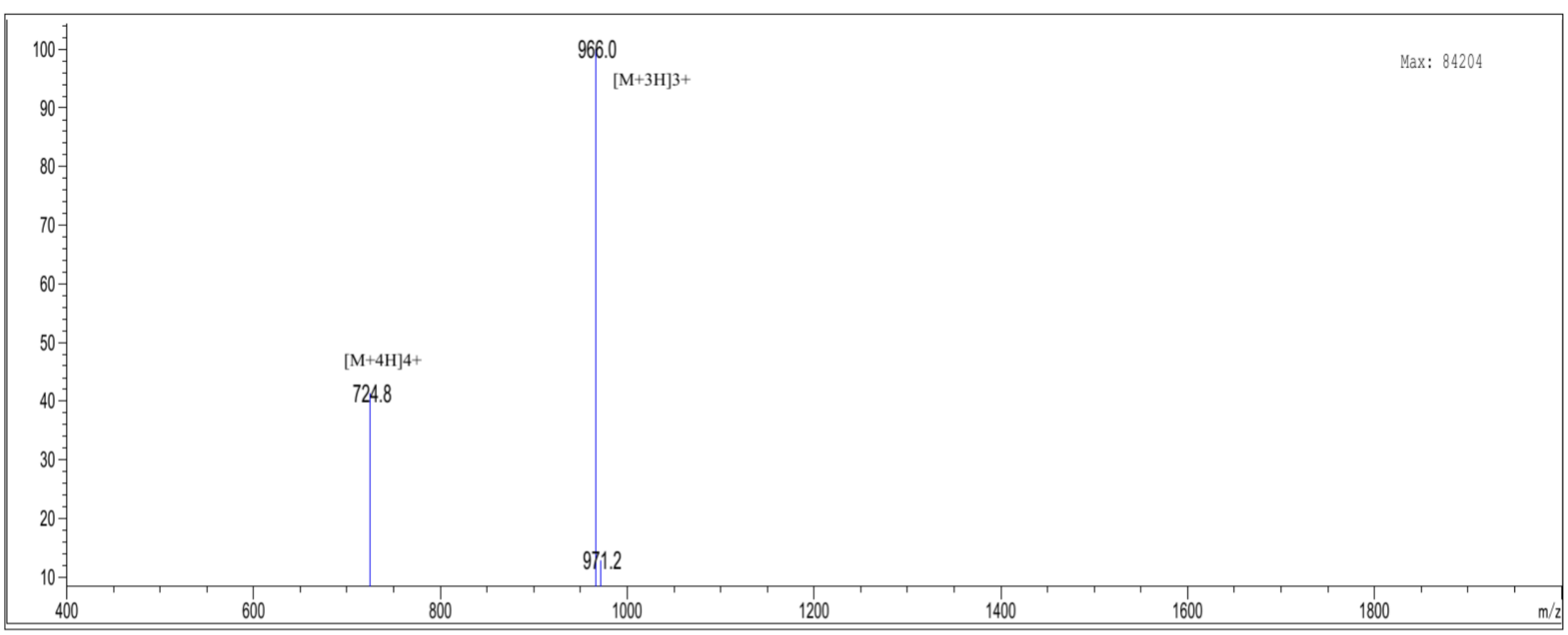

Supplementary Figure 3.1. Mass spectrometry analysis of Cy5-LANA(1-22) peptide. Agilent-6125B Instrument, 1.5L/min Nebulizer gas flow, -20.0v CDL, +4.5kv probe bias, 1.5kv detector, 0.2ml/min T. flow, B. conc.: 50%H2O/50%ACN

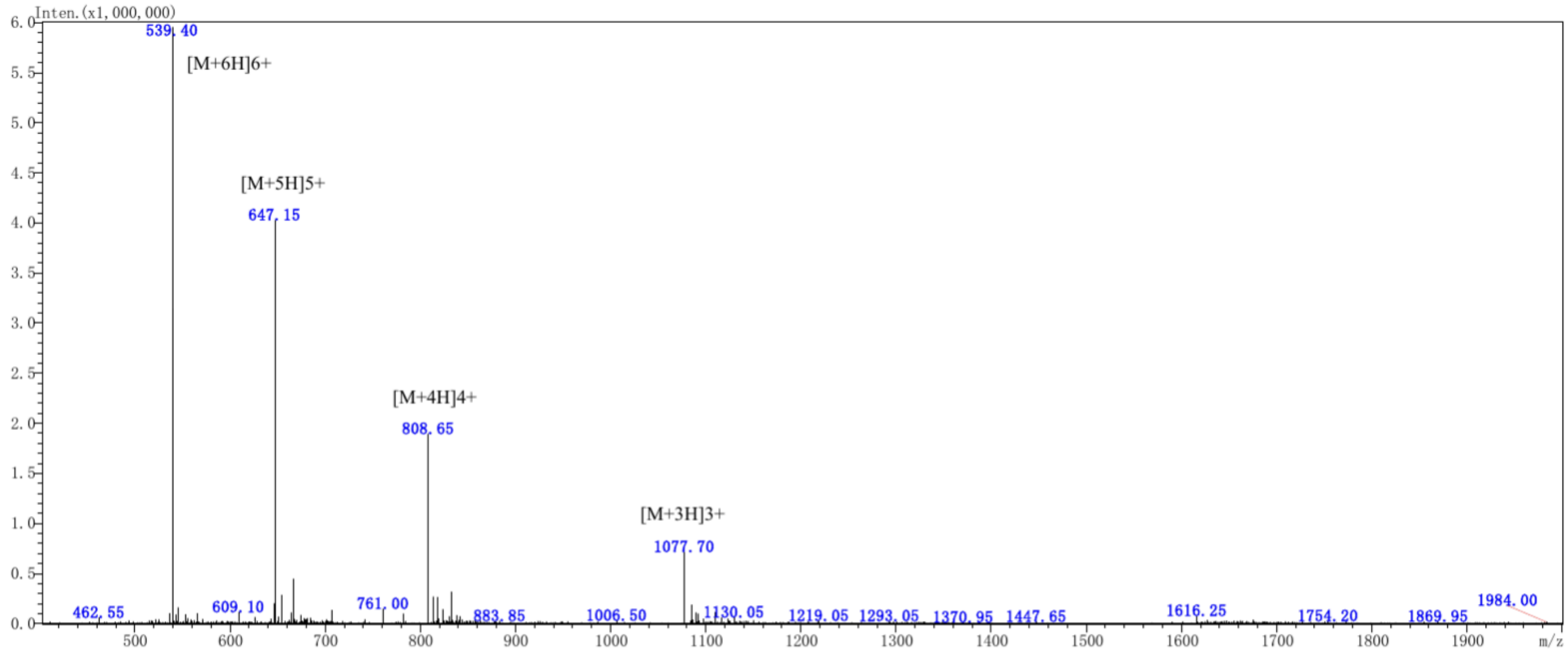

Supplementary Figure 3.2. Mass spectrometry analysis of CK25 peptide. SHIMADZU LCMS-2020 Instrument, 1.5L/min Nebulizer gas flow, -20.0v CDL, +4.5kv probe bias, 1.2kv detector, 0.2ml/min T. flow, B. conc.: 50%H2O/50%ACN

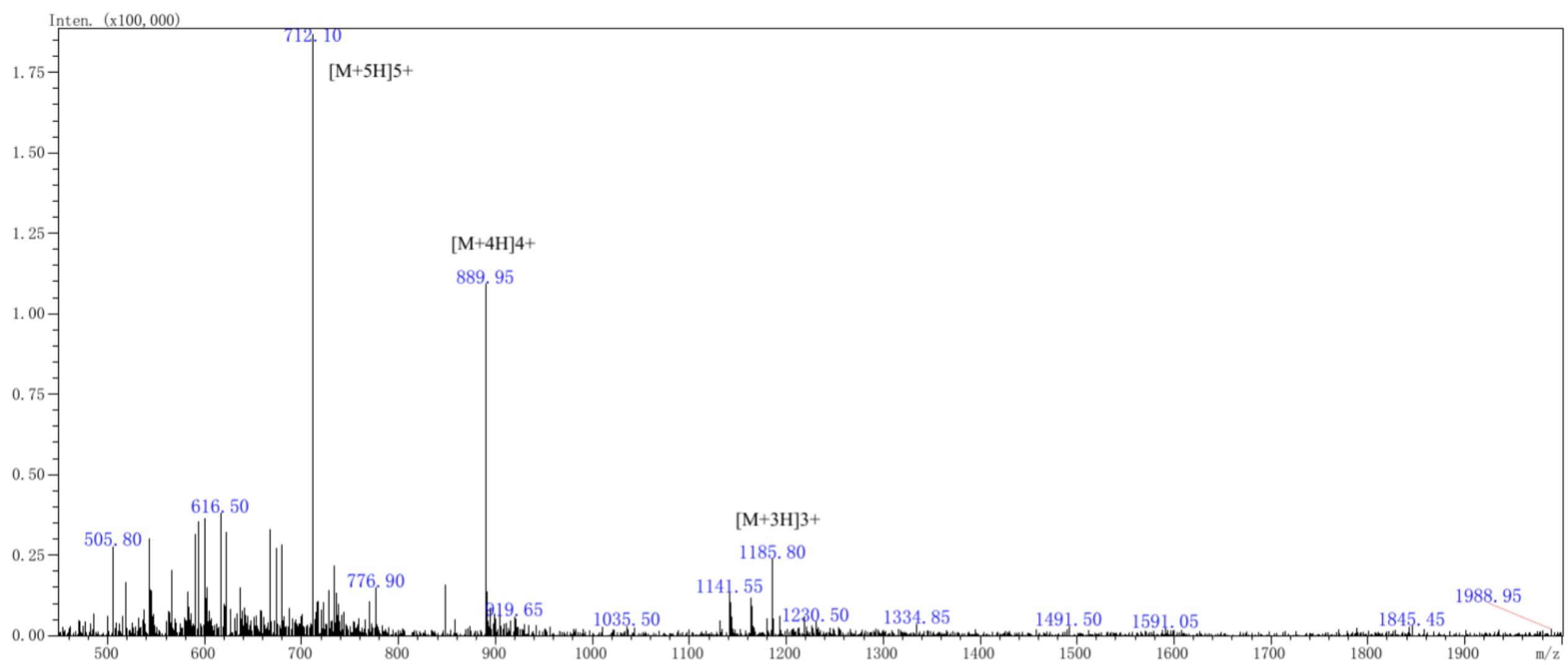

Supplementary Figure 3.3. Mass spectrometry analysis of CE27 peptide. SHIMADZU LCMS-2020 Instrument, 1.5L/min Nebulizer gas flow, -20.0v CDL, +4.5kv probe bias, 1.2kv detector, 0.2ml/min T. flow, B. conc.: 50%H2O/50%ACN

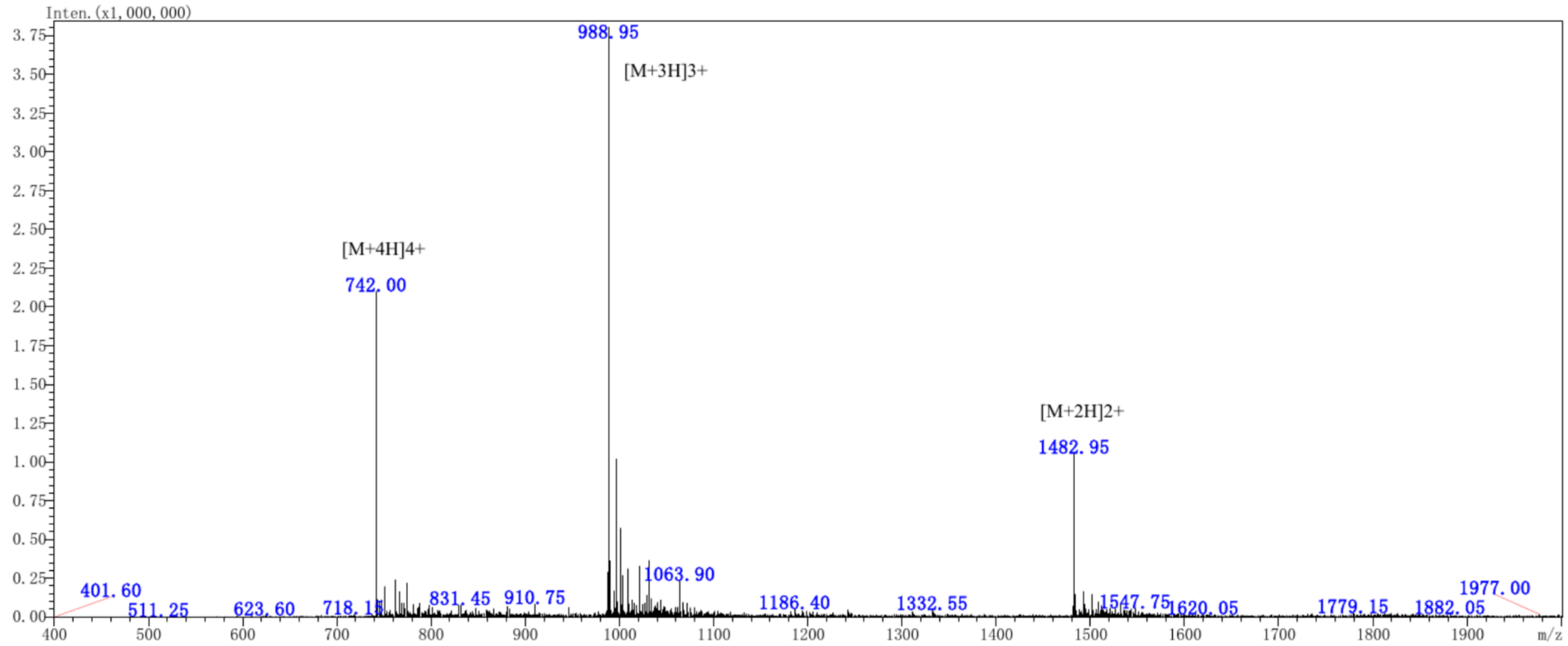

Supplementary Figure 3.4. Mass spectrometry analysis of CE23 peptide. SHIMADZU LCMS-2020 Instrument, 1.5L/min Nebulizer gas flow, -20.0v CDL, +4.5kv probe bias, 1.2kv detector, 0.2ml/min T. flow, B. conc.: 50%H2O/50%ACN

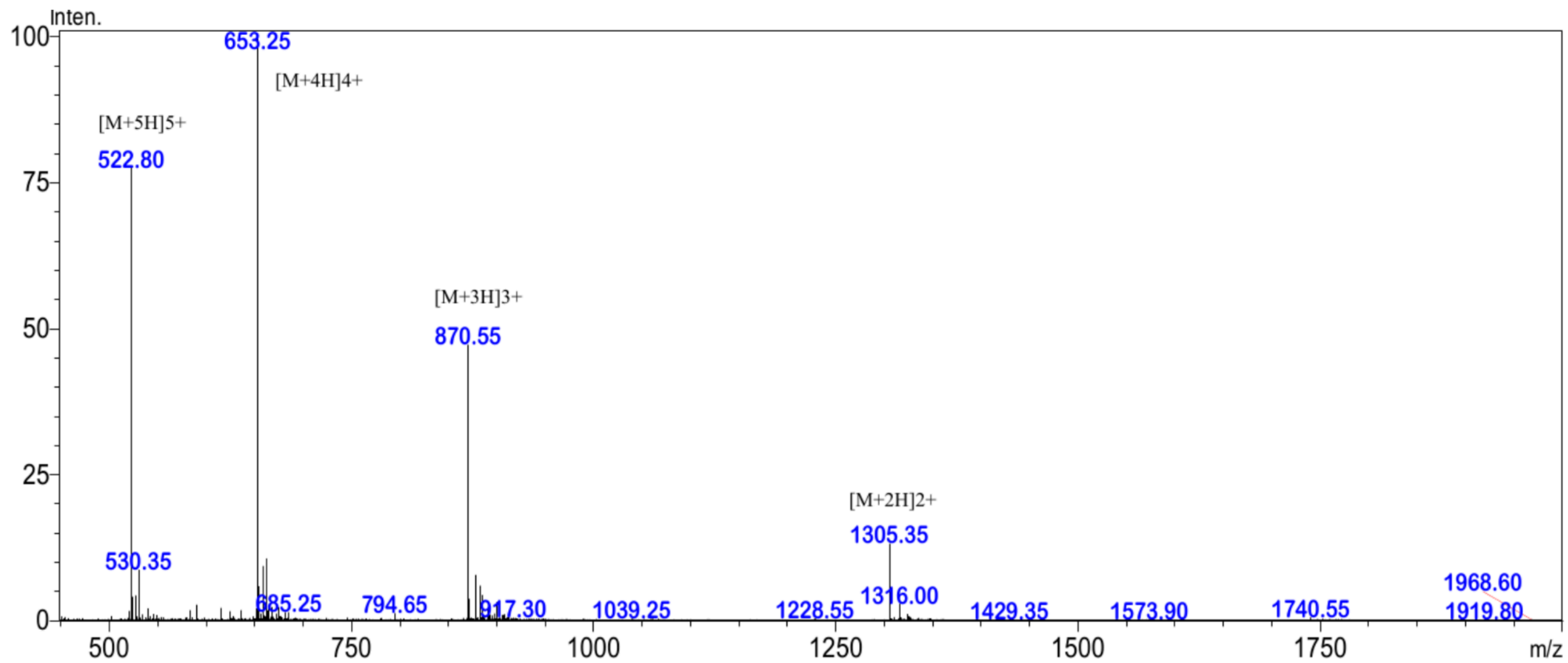

Supplementary Figure 3.5. Mass spectrometry analysis of PC20 peptide. SHIMADZU LCMS-2020 Instrument, 1.5L/min Nebulizer gas flow, -20.0v CDL, +4.5kv probe bias, 1.2kv detector, 0.2ml/min T. flow, B. conc.: 50%H2O/50%ACN

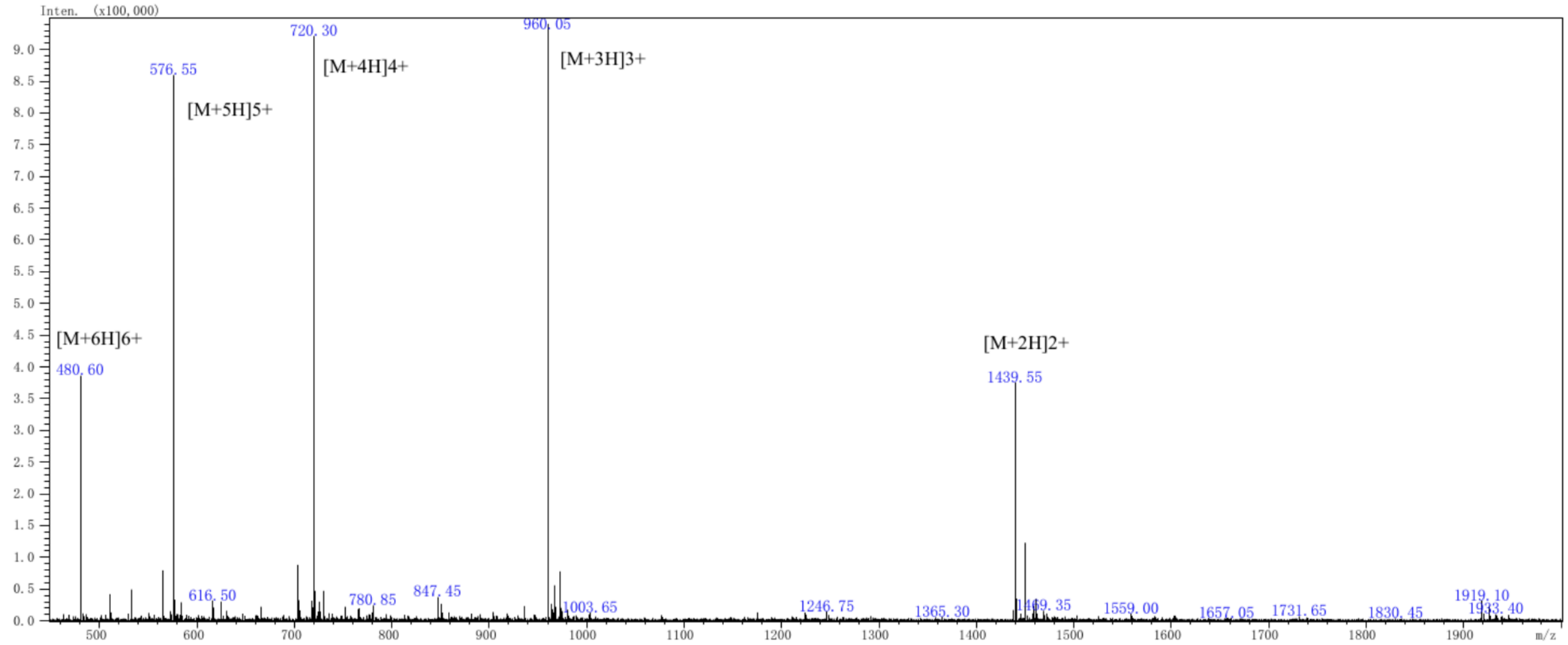

Supplementary Figure 3.6. Mass spectrometry analysis of CK21 peptide. SHIMADZU LCMS-2020 Instrument, 1.5L/min Nebulizer gas flow, -20.0v CDL, +4.5kv probe bias, 1.2kv detector, 0.2ml/min T. flow, B. conc.: 50%H2O/50%ACN

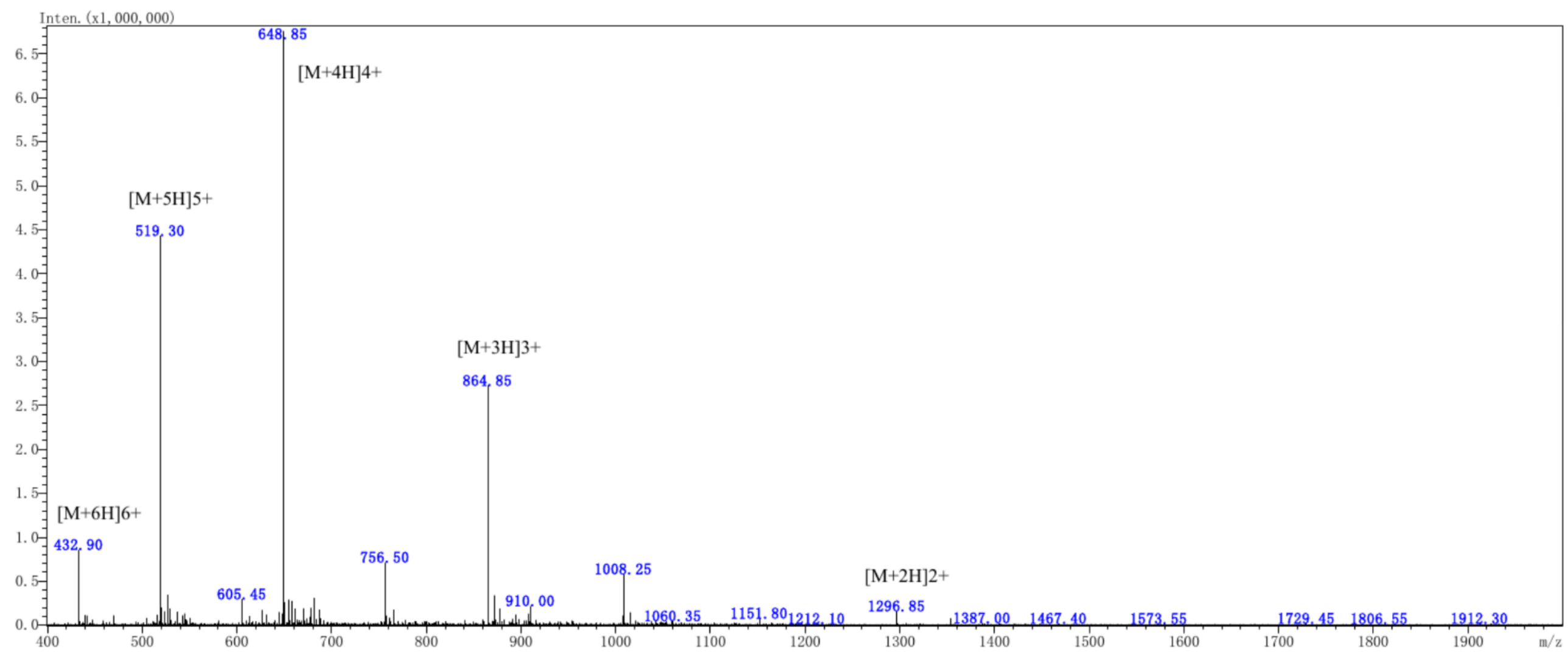

Supplementary Figure 3.7. Mass spectrometry analysis of DC21 peptide. SHIMADZU LCMS-2020 Instrument, 1.5L/min Nebulizer gas flow, -20.0v CDL, +4.5kv probe bias, 1.2kv detector, 0.2ml/min T. flow, B. conc.: 50%H2O/50%ACN

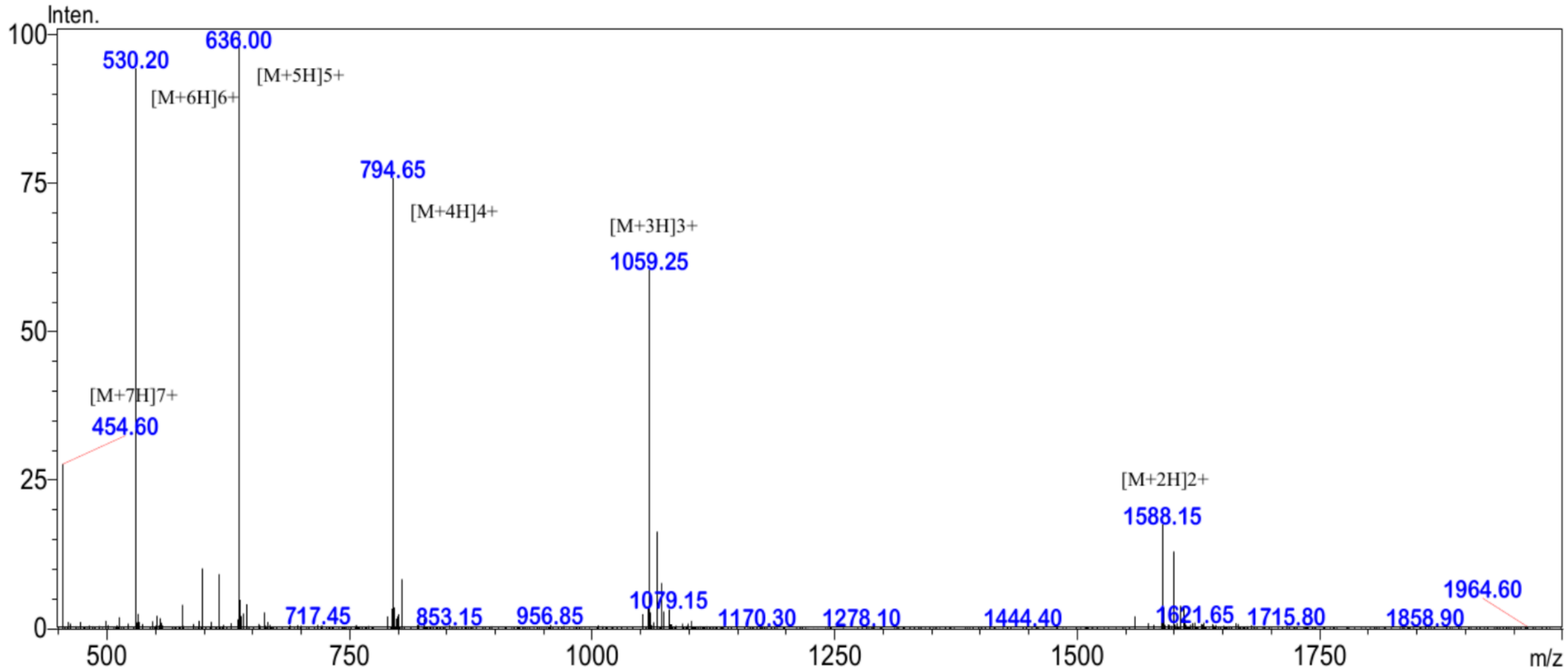

Supplementary Figure 3.8. Mass spectrometry analysis of CER25 peptide. SHIMADZU LCMS-2020 Instrument, 1.5L/min Nebulizer gas flow, -20.0v CDL, +4.5kv probe bias, 1.2kv detector, 0.2ml/min T. flow, B. conc.: 50%H2O/50%ACN

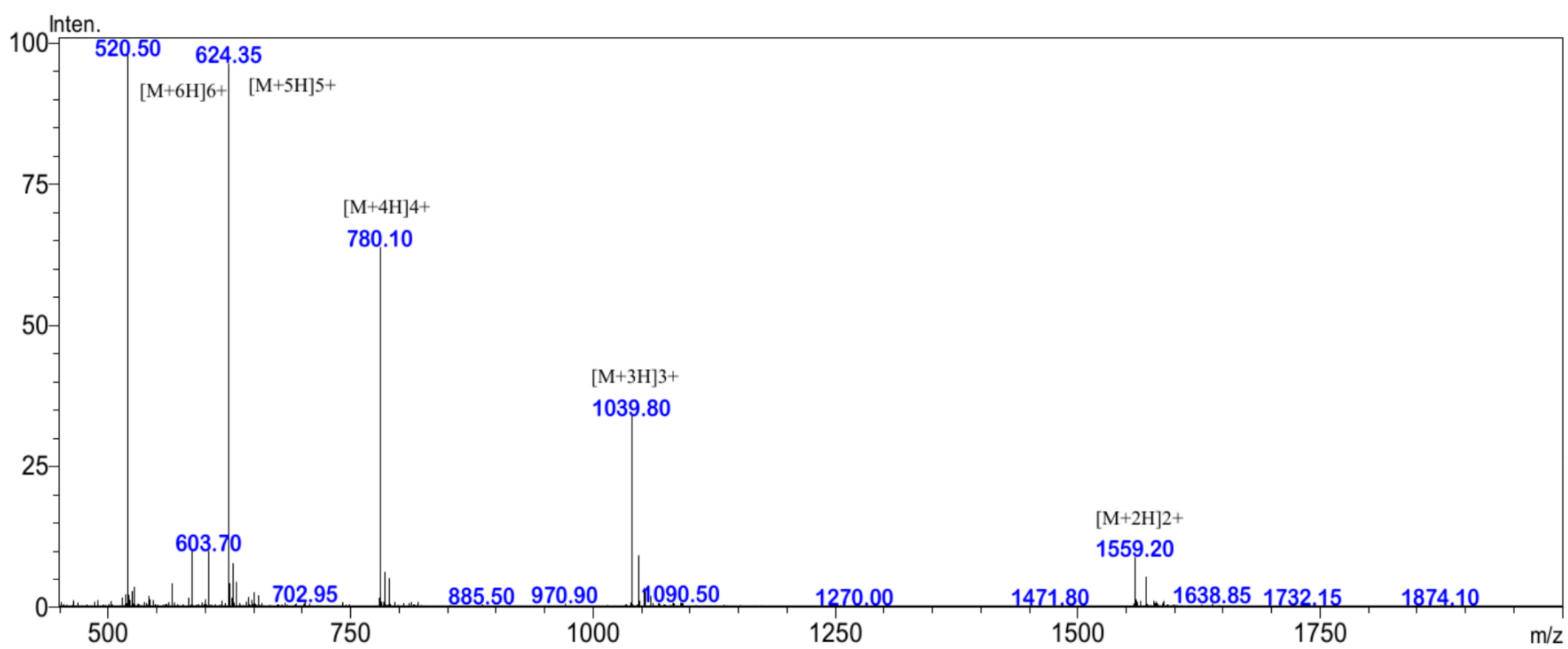

Supplementary Figure 3.9. Mass spectrometry analysis of CAR25 peptide. SHIMADZU LCMS-2020 Instrument, 1.5L/min Nebulizer gas flow, -20.0v CDL, +4.5kv probe bias, 1.2kv detector, 0.2ml/min T. flow, B. conc.: 50%H2O/50%ACN

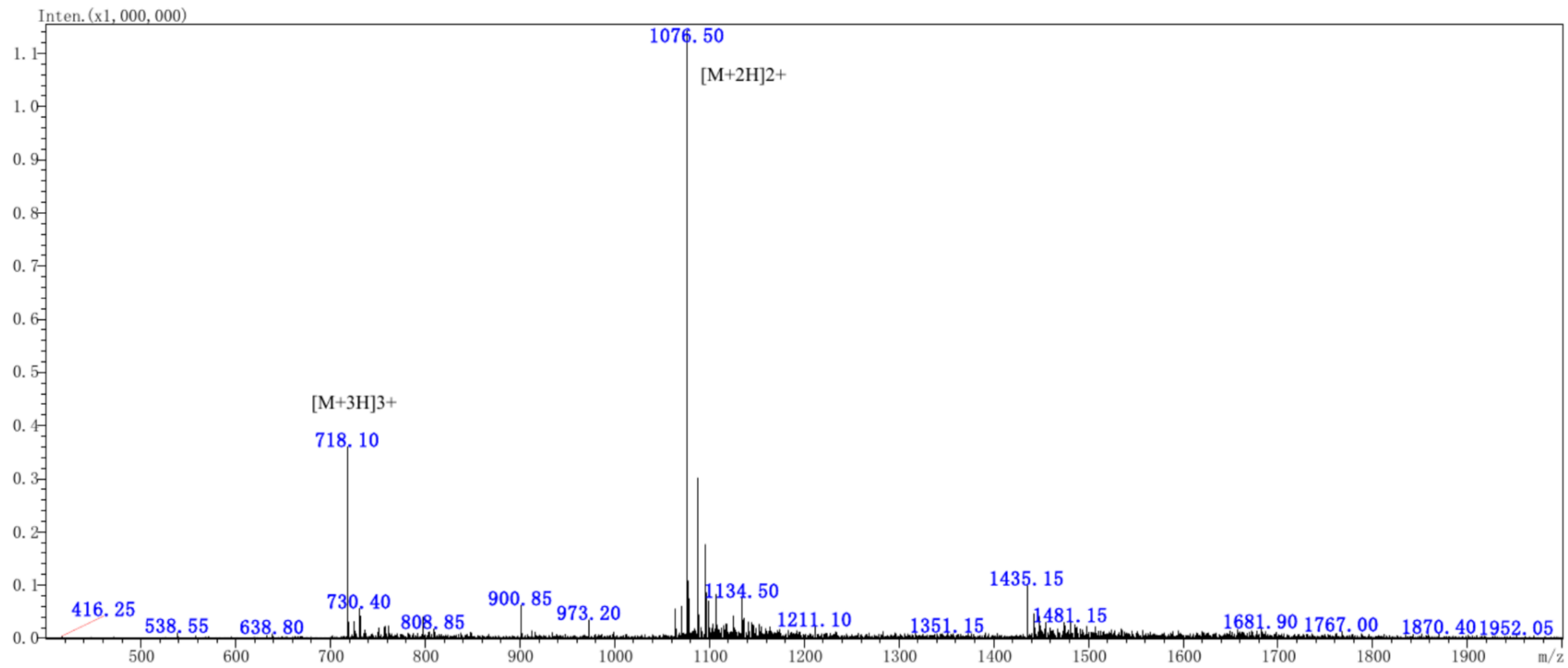

Supplementary Figure 3.10. Mass spectrometry analysis of MC16 peptide. SHIMADZU LCMS-2020 Instrument, 1.5L/min Nebulizer gas flow, -20.0v CDL, +4.5kv probe bias, 1.2kv detector, 0.2ml/min T. flow, B. conc.: 50%H2O/50%ACN

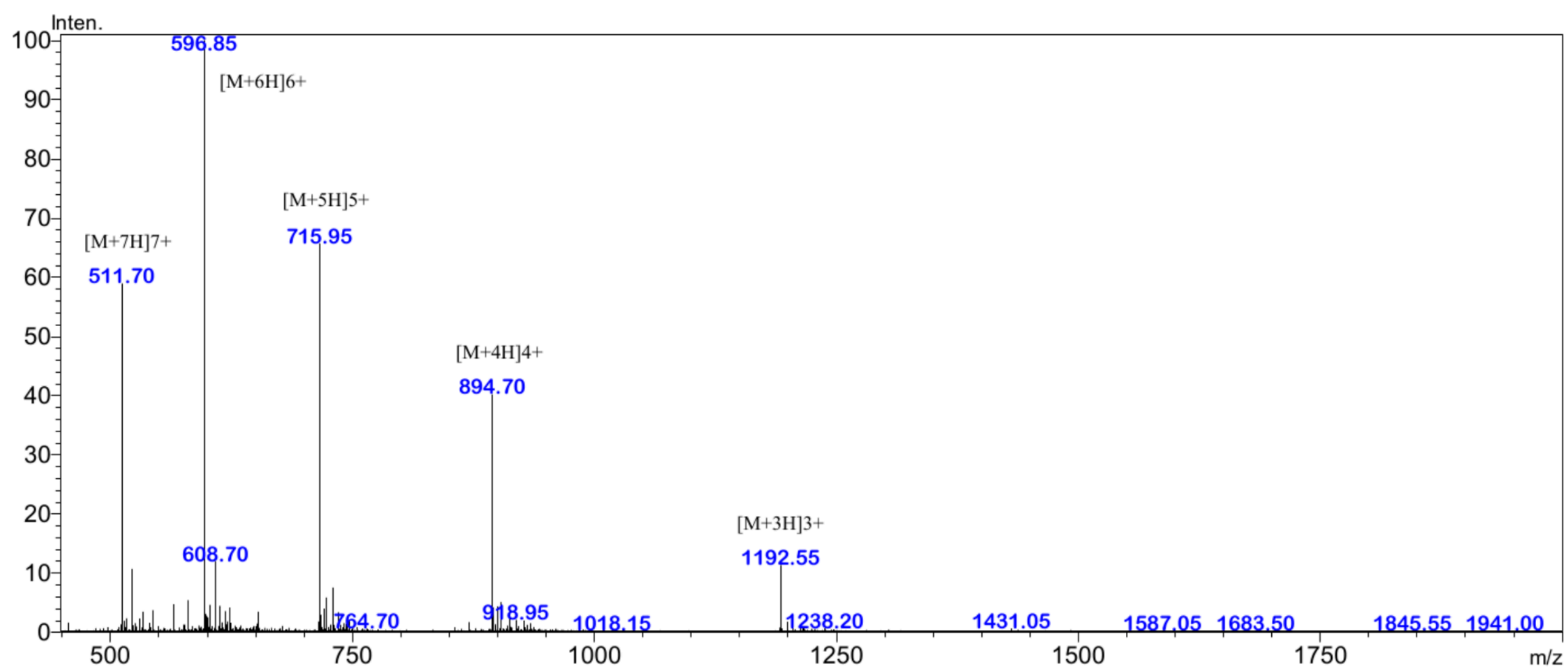

Supplementary Figure 3.11. Mass spectrometry analysis of CR26 peptide. SHIMADZU LCMS-2020 Instrument, 1.5L/min Nebulizer gas flow, -20.0v CDL, +4.5kv probe bias, 1.2kv detector, 0.2ml/min T. flow, B. conc.: 50%H2O/50%ACN

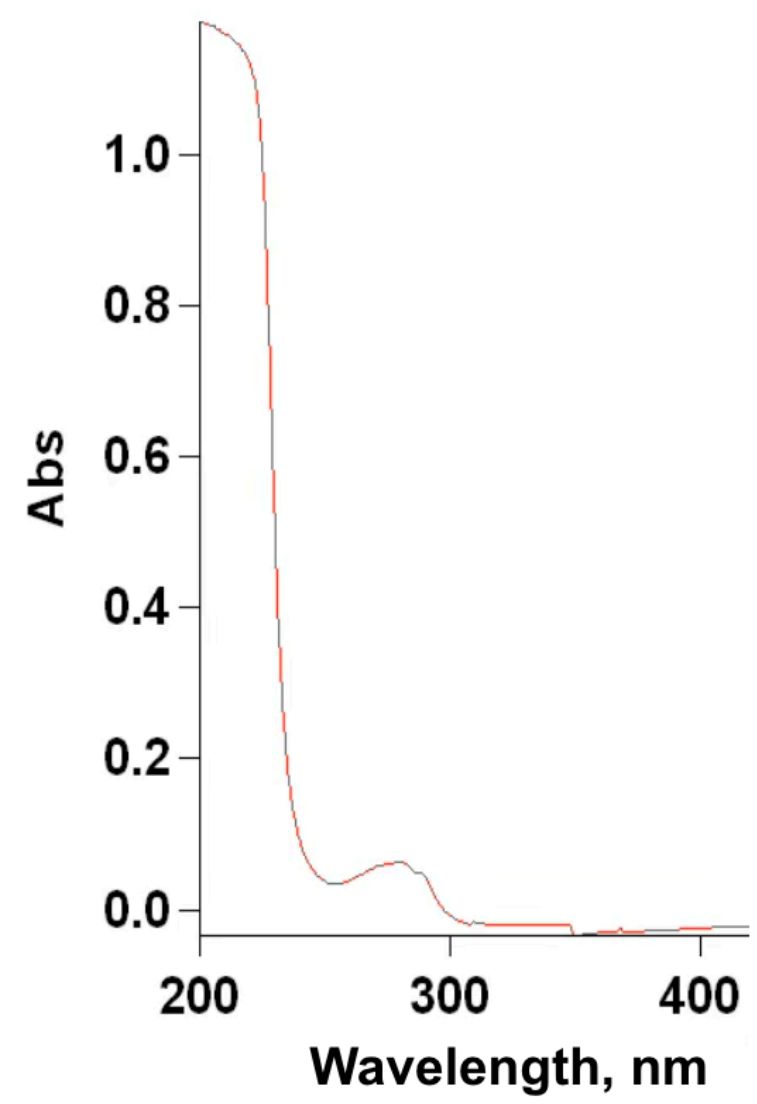

Supplementary Figure 4. Absorbance spectrum of DC21 peptide.

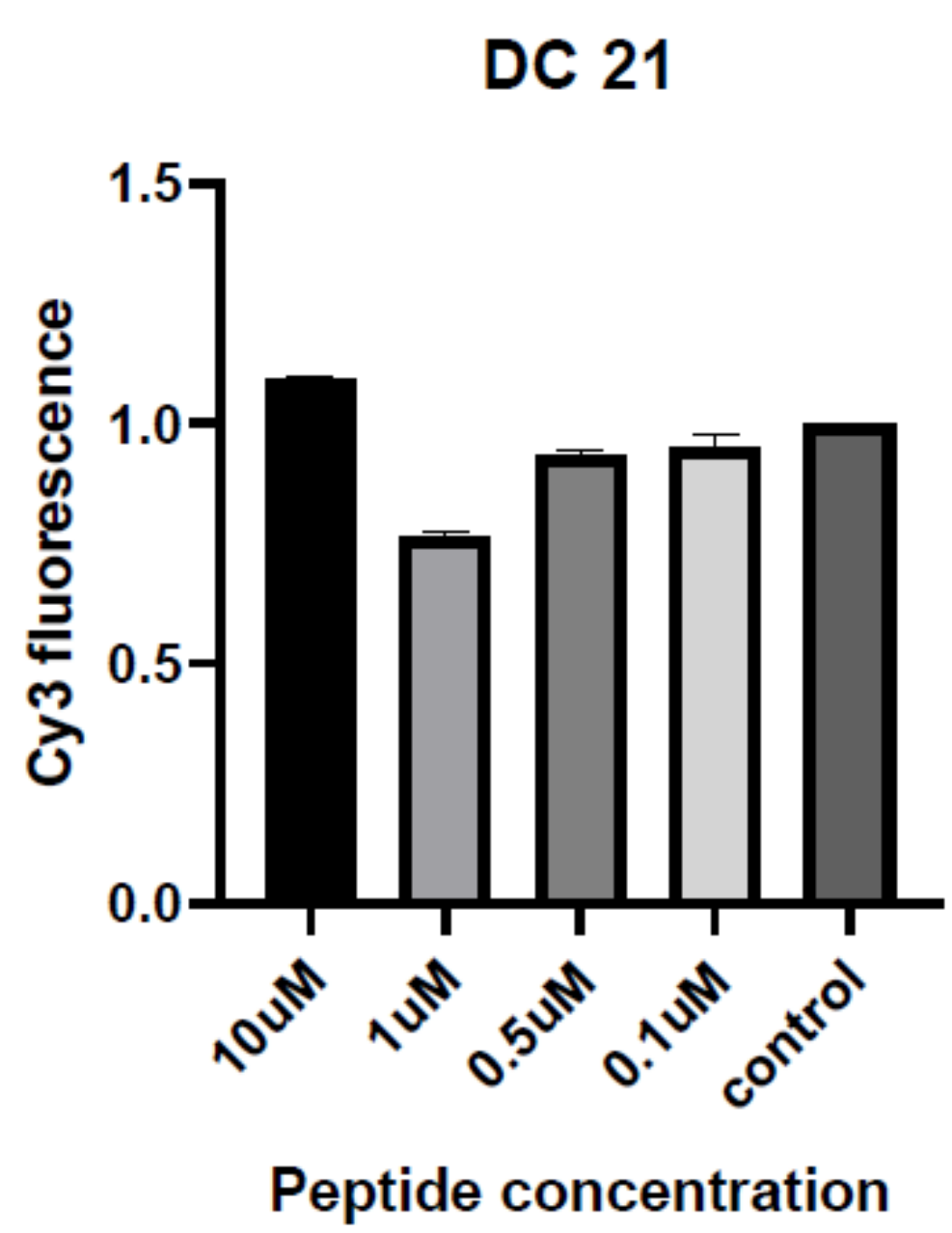

Supplementary Figure 5.1. Cy3 label fluorescence intensity in the presence of DC21 peptide

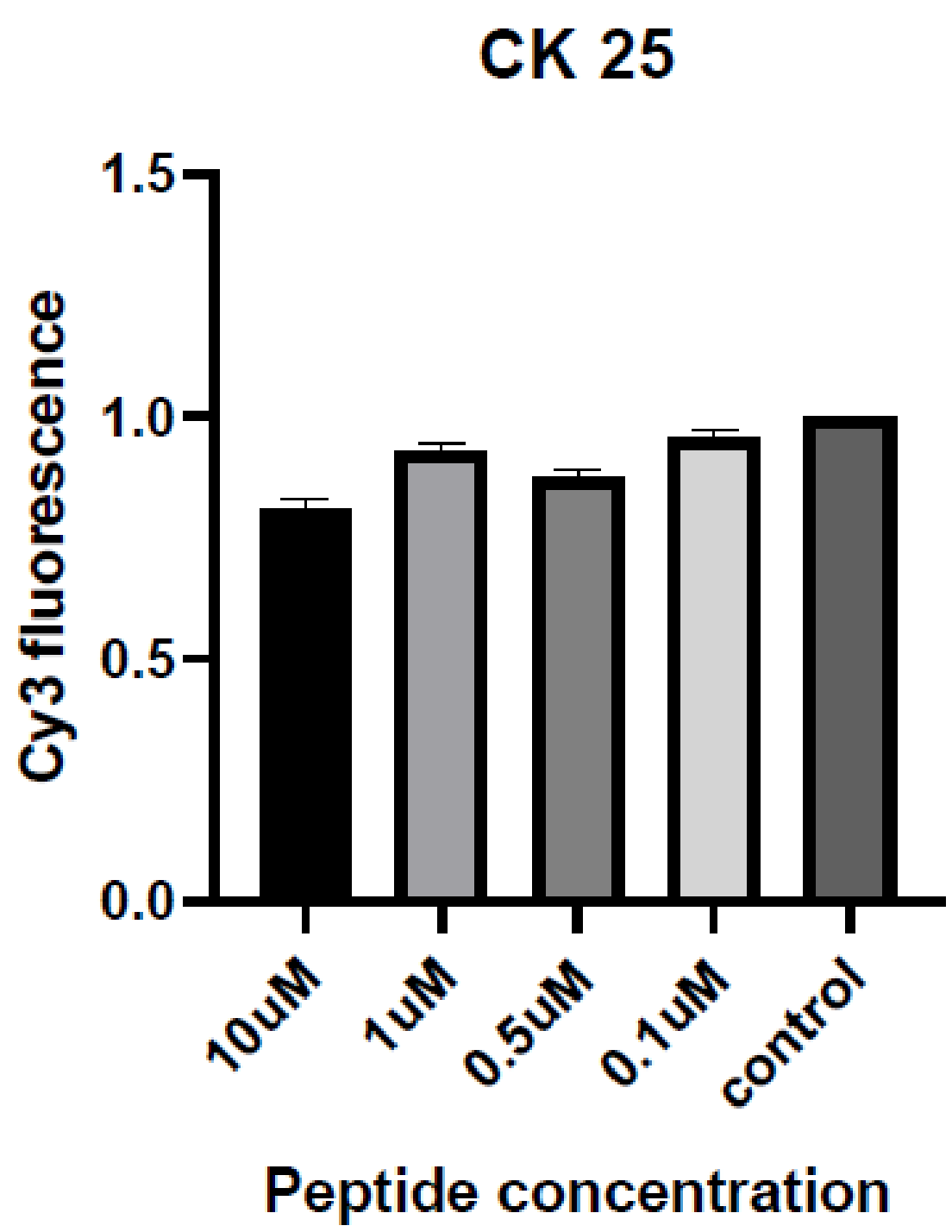

Supplementary Figure 5.2. Cy3 label fluorescence intensity in the presence of CK25 peptide

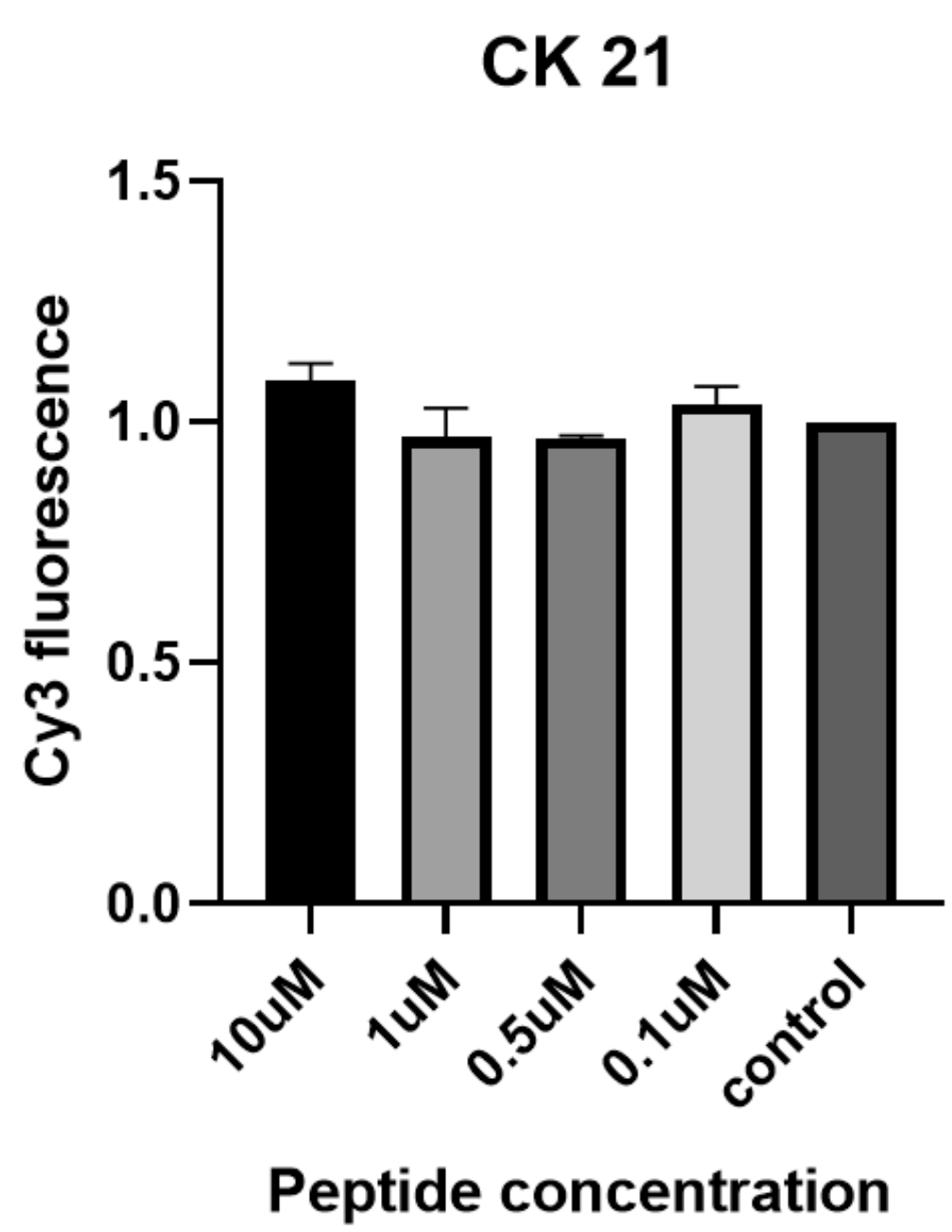

Supplementary Figure 5.3. Cy3 label fluorescence intensity in the presence of CK21 peptide

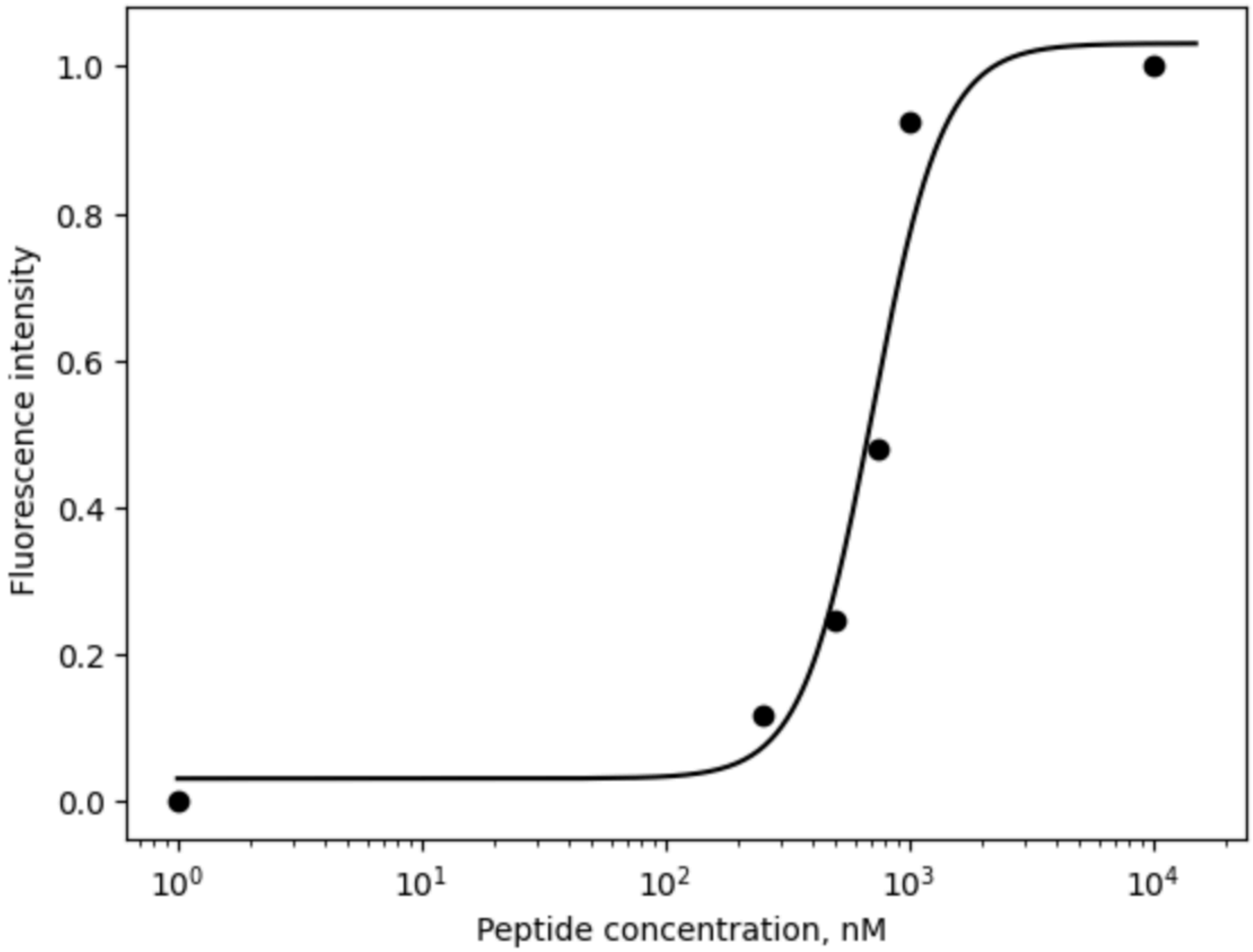

Supplementary Figure 6. Results of measurements of the dissociation constants for complexes of NCPs with unlabeled LANA peptide using the competitive binding protocol.

Peptide	Designed peptides aligned by anchor arginine
CK25	-CSDVIERWKSEEGKERRRRRLEKRK----------
MC16	-----------------MSEREIRWKRIKERMC---
CR26	-------CSMYEWLRQRHWERQERRRAEEARRR---
CE27	CSLEEWKQWGNERLKKMLEERRKYREE---------
CE23	-----CMNEIVKRVMENWKKRQEEKKKE--------
DC21	--------------DRRSLSRGERWALFRARRAEC-
PC20	----------------PRNSRTERFEAFQKWREHMC
CK21	------------CLSEYRKERYERFKKNWEKRK---
CAR25	---CSTNEVVRERIQAMLAKRKEWRKKR--------
CER25	---CSTNEVVRERIQEMLAKRKEWRKKR--------

PDB ID	Sequences of Interactive Structures aligned by anchor arginine	Closest design	Resolved	Similarity	PDB entries with compatible sequences of interacting proteins
3MVD	GCAEQGQLGRLSERSISGEGRRGKRDLLRPTQLIIT	CK25	25/25	27%	3MVD
8UX1	GCGEQGQLGRVPELFANRGGRQGLERLLVPKCVMLK	CK25	25/25	24%	8UX1
9M76	DNPKERGFWYDAEISRKRETRTARELYANVVLGDDS	CK25	25/25	23%	9M76,9M77
4KUD	QVIITDDQGRVIDDNNRRRSRKRGGENVFLKRISDG	CK25	25/25	21%	4KUD
8T3W	AIKTCGHVFCENCCKERLAARMRKCPTCNKAFSSND	CK25	25/25	21%	8T3W,8IEG,8T3Y,8T3T
8H1T	CQGPVYGFIFLFKWIEERRSRRKVSTLVDDTSVIDD	CK25	25/25	20%	9U5U,8H1T
7JOA	PRIELQEYYETGAFYLVKFKRIPRGNPLSHFLEGEV	CK25	25/25	19%	7JOA,7JO9,6X5A,7A08,…(+2)[1]
8DK5	ELSSLTSEDSAVYYCARKSSRLRSTLDYWGQGTSVT	CK25	25/25	17%	8DK5,7K60,8TOF,8SYP,…(+23)[2]
9ZEN	MNSLKSEDTAVYYCHVQEYGRVWSDHWGQGTQVTVS	CK25	25/25	12%	9ZEN
7BY0	EI-RQKIVLPSN-PNVRRTKRIRLKPLEYWRGERVT	CK25	23/25	20%	7BXT,7BY0
4JJN	GWQVIITDDQGRVI----RRRSRKRGGENVFLKRIS	CK25	21/25	29%	4JJN
7R5R	------------TSTVTKSRRISRRPSDWWVVKS--	CK25	14/25	30%	7R5R,6MUP,7YYH,6SEE,…(+5)[3]
7VDV	------------EKMFGRGSRHRKEVDYSDS-----	CK25	14/25	29%	7VDV,9WBZ,7VDT
9N6K	------------KIPTRFSNRQNKTVNYNIDYSDDD	CK25	14/25	23%	9N6K,9N6I,9GD2,9N6H,…(+4)[4]
7XPX	--------------PVRRSSRKSKAELQSEERKRID	CK25	12/25	36%	7XPX,7D1Z,7D20
7Y7I	--------------IYTRSGRLVKPPLSFWCGEREF	CK25	12/25	32%	7Y7I,9EAR
4X23	---------------VRRSNRIRLKPLEYWRGERID	CK25	11/25	35%	4X23
6QLD	---------------LRKSTRVKVAPLQYWRNEKIV	CK25	11/25	26%	6QLD,7ON1
8Q3X	---------------PGARLRSGRSTGAP-------	CK25	11/25	22%	8Q3X,8Q3M
8X19	----------------RKSMRQSTAEHTRQTFLRVQ	CK25	10/25	27%	9CA7,8X1C,8X19,9CAA,…(+1)[5]
9D3L	--------------PPRRSSRLT-------------	CK25	9/25	43%	9FGQ,9D3L,9D3K
7VVZ	-----------------FRHRKISVK----------	CK25	9/25	34%	7VVZ,7VVU
9EH2	-----------------RTRNEMTAEEKKRRAHQKE	CK25	8/25	38%	9EH2
6UH5	------------------SSRLNRVFQRRFQQDIEA	CK25	8/25	28%	6UH5
6GEN	-------------------SRPVKRINYSLADLEAR	CK25	7/25	44%	6GEN,6GEJ
7UV9	----------------RYSQRL----RRRY------	CK25	6/25	52%	7UV9
9E3D	-----------------RGARQK------SVPVIRH	CK25	6/25	38%	9E3D,9E0R,9E3U,9E09
9D3M	----------------RRSAR---------------	CK25	5/25	60%	9D3M
4R8P	TALRSGNKECPTCRKKLVSKRSLRPDPNFDALISKI	MC16	16/16	25%	4R8P,8PP7,9DGG,9DBY,…(+2)[6]
8HQY	-------------HAWTHRLRERKQLVIYEEISDPE	MC16	16/16	25%	8HR1,8HQY
8UW1	----GSRGRLRGGLGWESSLRQRPMPRLTFQAGDPY	MC16	16/16	22%	8QZM,8U5H,8UW1
7JZV	LLNQKKGPSQCPLCKNDITKRSLQESTRFSQLVEEL	MC16	16/16	16%	7JZV,7LYB
8OL1	PRIQLEEYSNTRAYYFVKFKRNPKENPLSQFLEGEI	MC16	16/16	16%	8OL1,7CCR,7CCQ,7C0M,…(+2)[7]
9T4V	QLVNLQKTLLEKASQEGRSLRNKGSVLIPGLVEGST	MC16	16/16	16%	7ENN,9T4V
7EGP	TPNLLQISAAELERLDKDKDRDTRRKRRQGRS----	MC16	15/16	38%	7EGP,7EG6
7X3V	FILRQFEQMKVQFADMKPGKRQLRRIQRQ-------	MC16	12/16	27%	7X3V
1ZLA	---------------PGMRLRSGRSTGAP-------	MC16	12/16	24%	1ZLA,5GTC
9QAJ	------------------SGRSVRAETRS-------	MC16	11/16	31%	9QAJ
8IHM	---------RRKIITSEGIERSFKNEH---------	MC16	10/16	24%	8IHM,8IHT
8PP6	RNSAEAFKCSICDVRKGTSTRKPRIN----------	MC16	9/16	31%	8PP6
7K6Q	YISTVMEDVDESLFSPAARGRR----PVKY------	MC16	9/16	30%	7K6Q,7K6P
8SVF	QSKCQGPVYGFIFLFKWIEERRSRR-----------	MC16	8/16	36%	8SVF
5KGF	----LTKAADISLDNLVEGKRKRRS-----------	MC16	8/16	27%	7YQK,5KGF
8KD7	VKTGSRGQYSDEND----TDRQ---------ITKLD	MC16	6/16	33%	8KD7
8TXX	VDKEQ---NRQKGSPDEYHLRA--------------	MC16	5/16	21%	8TXV,8TXW,8TXX
6ZHY	-------------------GRSL-------------	MC16	4/16	37%	6ZHY,6ZHX
9IPU	TVEKASLCCPFCRRRVSSWTRYHTRRNSLVNVELWT	CR26	26/26	29%	9IPU,8SMX,8SNA,8SN6,…(+19)[8]
8GUJ	VLTKCFHVFCFECVRGRYEARQRKCPKCNAAFGAHD	CR26	26/26	25%	8GUJ
8GUI	VLTKCFHVFCFECVKTRYDTRQRKCPKCNAAFGAND	CR26	26/26	23%	8GUI,9KQ0,8IEJ
9RL4	CPLLETLTDAEMEKKIRDQDRNTRRMRRLANT----	CE27	27/27	25%	9RL4,7Y8R
9GFM	GELDDNGDLKKEINERLLTARQRALLQKARS-----	CE27	27/27	21%	9GFM,9GF6,9GEV,9GFB
7E9C	WEIITTDEQGNIIDGGQKRLRRRGAKTEHYLKRSSD	CE27	27/27	19%	6OM3,7E9C,7E9F
9NH8	RLEEEERQKELEEIYMLPRMRN--------------	CE27	22/27	25%	9NH8
9L22	--------------LIVEGKREKKKVERLTMQVSSL	CE27	13/27	29%	9L22,9L1X,8KCY,8KD1
9WC0	---------------VRAETRSRAK-----------	CE27	10/27	27%	9WC0
3TU4	DGWQVIITDDQGRVIDDNNRRRSRKRGGENVFLKRI	CE23	23/23	25%	3TU4,4LD9
8JHG	FKS------PAPVINSKYGLRETDKRLNRL------	CE23	19/23	21%	8T9H,8JHG
9JNW	DFKK----------LLNPTKRERKENYSIDNY----	CE23	14/23	29%	9JNX,9JNW,9JNV,7X3X,…(+1)[9]
9Y4P	--------------------R--------SVPVIRH	CE23	1/23	100%	7TAN,9Y4P,9FH9
9SLJ	-------------APRKGSSRVAKTNSLRRRKLASF	DC21	21/21	27%	9SLJ,9SI9,9SJ5,8RUQ,…(+2)[10]
7E8I	HHPKDNLIKLVTAGGGQILSRKPKPDSDVTQTINTV	DC21	21/21	15%	7E8I,9UX9,7LYC
6VEN	----------------VSSSRDNRASNRRFQQDIEA	DC21	19/21	26%	6VEN
9B1E	-----LNAETLLLNTRRTSKRSSVMENTMKVYEKLS	PC20	20/20	21%	9B1E
8F86	GTMGLKATGRLCTVAKARGLRACRGELRDTILDWED	PC20	20/20	19%	8F86,8G57,8OF4,9EIL
8Q3E	---------------GGYNLRPRTYQPQRYGGG---	PC20	17/20	18%	5MLU,8Q3E,8Q36
5E5A	-----------GKSTHPMVTRSKAD-----------	PC20	9/20	31%	5E5A
9F00	---------------AEYNLRSR-------------	PC20	7/20	32%	9F00
6PA7	NRIFGFPVHYTDVSNMGRGARQKLLGRSWSVPVIRH	CK21	21/21	22%	9E2Q,9E00,9E2R,9E05,…(+1)[11]
6TDA	CPRVEILTKEEIQKREIEKERNLRRLKRETDR----	CK21	20/21	31%	6TDA
8SKZ	AHLG------IELSPTGRPKRRTRKSINYSK-----	CK21	19/21	27%	8SKZ
8AV6	RLAKLNQLKERERLKADLGNRKSLASQIRMKNIANL	CAR25	25/25	29%	8AV6
9IHE	MVQIITYRDYLPLVLGPTAMRKYLPTYRSYNDSVDP	CAR25	25/25	21%	9IHE,9GEP,9GEN,9IHF,…(+2)[12]

Additional PDB entries with compatible sequences of interacting proteins:

[1] 6X59,6XJD

[2] 7U0J,6DZT,8UBJ,6E0P,8EVG,7K78,8EVJ,7U0I,7U0G,8VFX,8UBL,7K5Y,8U9S,8SPU,6E0C,7K63,8SPS,8EVI,7K61,8UBK,8EVH,7K5X,8UBI

[3] 7YWX,6SEF,6SE6,6MUO,7PII

[4] 9R5S,9GD1,9GD3,7TN2

[5] 9CA8

[6] 8GRM,9DDE

[7] 6Y5D,6Y5E

[8] 8SMZ,8SMY,8X7K,8SN5,8SN3,8UPF,8SN9,8SN7,8SN4,8SMW,8U13,8SN0,8X7J,8U14,8SN8,8SN2,9KQ2,8SN1,8X7I

[9] 9LJ2

[10] 9SI3,8RUP

[11] 6PA7

[12] 9GEQ,9IHD

Supplementary Figure 7. Comparison of designed peptides with known nucleosome acidic-patch-binding sequences

References

Kuipers, B. J. H., and Gruppen, H. (2007). Prediction of molar extinction coefficients of proteins and peptides using UV absorption of the constituent amino acids at 214 nm to enable quantitative reverse phase high-performance liquid chromatography-mass spectrometry analysis. *J. Agric. Food Chem.* 55, 5445–5451. doi: 10.1021/jf070337l

Oleinikov, P. D., Fedulova, A. S., Armeev, G. A., Motorin, N. A., Singh-Palchevskaia, L., Sivkina, A. L., et al. (2023). Interactions of Nucleosomes with Acidic Patch-Binding Peptides: A Combined Structural Bioinformatics, Molecular Modeling, Fluorescence Polarization, and Single-Molecule FRET Study. *Int. J. Mol. Sci.* 24, 15194. doi: 10.3390/ijms242015194
